# Virgin Olive Oil Phenols, Fatty Acid Composition and Sensory Profile: Can Cultivar Overpower Environmental and Ripening Effect?

**DOI:** 10.3390/antiox10050689

**Published:** 2021-04-27

**Authors:** Maja Jukić Špika, Slavko Perica, Mirella Žanetić, Dubravka Škevin

**Affiliations:** 1 Institute for Adriatic Crops and Karst Reclamation, Put Duilova 11, 21000 Split, Croatia; Slavko.Perica@krs.hr (S.P.); Mirella.Zanetic@krs.hr (M.Ž.); 2 Centre of Excellence for Biodiversity and Molecular Plant Breeding (CoE CroP-BioDiv), Svetošimunska cesta 25, 10000 Zagreb, Croatia; 3 Faculty of Food Technology and Biotechnology, University of Zagreb, Pierottijeva 6, 10000 Zagreb, Croatia; dskevin@pbf.hr

**Keywords:** phenols, fatty acids, C18:1/C18:2, growing conditions, sensory quality, maturity index, *Olea europaea* L.

## Abstract

The authenticity and typicity of monocultivar oils and knowledge of the changes that environmental olive growing conditions bring to naturally present antioxidants and sensory attributes of virgin olive oils (VOO) are important for quality and safety improvement. This study delivers a comprehensive evaluation of the factors affecting phenolics, fatty acid composition and sensory characteristics of cultivars Oblica and Leccino VOOs throughout ripening season at two distinct olive growing environments during three consecutive crop years, and ranks the importance of each factor. Specified parameters were significantly influenced by olive growing environmental conditions. At the colder location of higher altitude, both cultivars gained higher amount of stearic, linoleic and linolenic fatty acids, as well as a higher proportion of phenolic compounds, but lower amounts of oleic fatty acid. At the warmer location of lower altitude, both cultivars had oils with lower level of fruitiness, bitterness and pungency. Analysis of the main components showed that VOOs were primarily differentiated by the cultivar, then main groups were divided with regard to the growing site, while harvest period affected the biosynthesis of natural VOOs antioxidants but had the least impact. These results reveal that the composition of fatty acids, phenolic content and sensory profile are predominantly characteristics of a cultivar.

## 1. Introduction

Derived from fruit of the olive tree (*Olea europaea* L.), extra virgin olive oil has a high amount of antioxidants that ameliorate the oxidative stress produced by free radicals and, subsequently, cellular damage [1]. Most of the beneficial effects of virgin olive oil (VOO) and its constituents are mediated by its phenolic compounds and α-tocopherol antioxidant activity [2,3]. The process of food acceptance or rejection by consumers is of a multi-dimensional nature [4] and, surely, sensory quality should be considered as a key factor guided by the fact that consumers seek food with certain sensory characteristics [5]. Safety, sensorial and organoleptic value, as well as nutritional and health promotion effects, define the quality of oil and, nowadays, such aspects of quality are closely related to either cultivar, or to the region of production, especially those owning PDO (Protected Designations of Origin) or PGI (Protected Geographical Indications) protection. 

From that standpoint, studies that evaluate the composition of virgin olive oil demonstrate that monocultivar oils differ from each other based on their compositional characteristics [6,7,8,9]. These findings are useful in meeting the sensory expectations of new VOO unaccustomed consumers by comprehensive characterization of the tight relationship of volatiles and phenolics with VOOs sensory attributes. However, olive production is expanding from traditional areas [10,11,12] and it can be expected that olive-rich areas will be changed even more, and/or that such changes will induce a distinct metabolite profile of single characterized VOOs. Differences in primary antioxidant content due to altitude and latitude and climatic parameters of a well-defined and restricted geographic area have been investigated on several occasions, but contrasting results have been found. Higher phenolic compound content was identified in Mastoides VOOs obtained at lower altitudes [13], while opposite results were reported for Chemlali, Chétoui [14] and Carolea VOOs [11]. The differences arise from the combined effect of temperature and sun intensity, and the availability of water in different soils [15]. Although it was hypothesized that higher temperature and sun intensity are possibly more favorable for phenolic accumulation in areas of lower altitudes, this obviously cannot be observed uniformly. The literature suggests that cultivars can presumably adapt differently to temperature variations and, moreover, can do so in combination with available water and other environmental factors [16,17], meaning multiple modifications and combinations are possible; therefore, it is obvious that ranking the importance of each factor affecting the accumulation of phenolic compounds is not yet fully understood. 

Olive oil is strongly characterized by its fatty acids composition [18], which has wide practical application [19,20,21,22,23,24], including quality appraisal and categorization [25], varietal characterization [14,26,27], and geographical origin classification [28]. Still, fatty acid composition is influenced by factors defining the site of cultivation. Studies have shown substantial deviations from the simple expectation that “the higher the altitude the higher the corresponding content of unsaturated/polyunsaturated/saturated fatty acids” [10,13,14,29]. Diverse patterns of changes were reported for fatty acid composition with climatic parameters [30,31,32]. 

The compositional diversity of VOOs, in addition to the above, is a reflection of variable technological steps [16,33,34,35,36,37]. Among them, stage of olive maturity is considered an extremely influential factor, and to know and control its effect on the final product is paramount. This complex system could be solved through the approach of observing the results of quantitative descriptive analysis that unites odor, flavor and retro-nasal attributes as result of VOOs’ numerous compounds and their synergistic and antagonistic actions.

Consequently, the present research aimed (i) to study, in depth, the changes of basic quality parameters, phenolic content, fatty acid composition and sensory proprieties of VOOs of two important cultivars at different fruit ripening stages, both grown in two distinct environments; typical olive growing coastal plain and hilly hinterland, a fringe growing site of olive cultivation; (ii) to correlate climatic conditions recorded in three consecutive crop years with investigated VOO parameters; and (iii) to determine the main sources of variation within the results using multivariate statistics and rank the importance of each factor studied.

## 2. Materials and Methods

### 2.1. Environmental Conditions of Two Olive Experimental Sites

Experiments on Oblica, an autochthonous, dominant Croatian cultivar, and Leccino, a well-adapted, introduced cultivar, were conducted in two olive orchards located in different olive subregions during three consecutive years (2010, 2011 and 2012). The first, Kaštela, is an experimental collection of the Institute for Adriatic Crops, and it is located 0.5 km from the coast (43°55′ N; 16°35′ E) and 28 m above sea level. It is influenced by the Mediterranean climate, defined as the Csa climate type [38]. The land there is an almost flat coastal plain and the effective soil depth is 75 cm. It is clay-loam with alkaline reaction, and with a low-to-medium level of skeleton. Some 50 km southeast, in the hilly hinterland behind the coastal mountains, is Šestanovac (43°27′ N; 16°55′ E), the second site. It is located 358 m above sea level and 7 km from the coast. According to the Köppen–Geiger classification, the climate type here is defined as Cfa [38], with the main influence of the continental climate in the winter and the Mediterranean climate in the summer. The soil of this olive orchard is obtained by stone crushing and grinding the surface layer up to 50 cm depth. It is moderately carbonate with alkaline reaction and a high level of skeleton.

**Figure 1:**
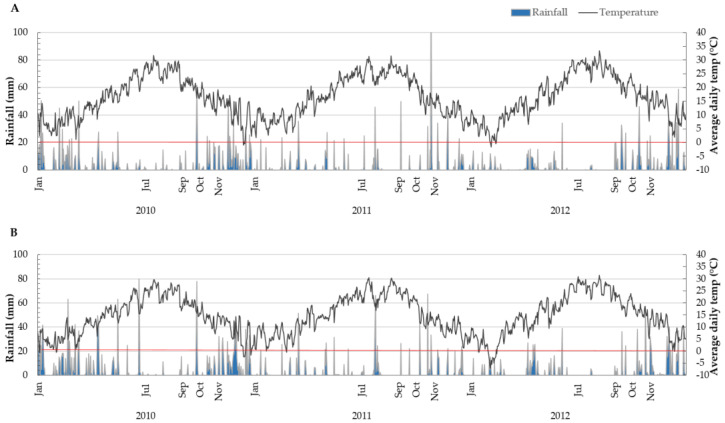
Microclimatic parameters measured for each olive growing site; (**A**) Kaštela and (**B**) Šestanovac.

Climate parameters (average daily temperature and rainfall) registered for the studied years at selected locations (obtained from the Meteorological and Hydrological Service of Croatia) show that Kaštela (Figure 1A) is associated with less rainfall and higher temperatures (daily mean higher from 0.9 to 2.6 °C during the olive fruit development and ripening period from July to November), indicating Kaštela as a more drought affected and warmer growing site in comparison to Šestanovac (Figure 1B). 

The year 2010 was recorded as the highest rainfall year (1397.9 mm, 1961.9 mm; Kaštela and Šestanovac respectively) and the year with the lowest average daily air temperatures. Comparing the overall 3 years of research for the period July–November, higher mean daily temperatures were recorded in 2012 (except September, the highest temperatures were measured in 2011 at both locations). 

### 2.2. Sampling of Olive Fruits, Olive Processing and Oil Extraction

In each crop year, 48 batches of olive fruits were generated from two experimental orchards, involving 2 cultivars and 4 harvest dates. Starting from the end of September, healthy olive fruits collected from all 4 sides of each of the 3 sampled trees were hand-harvested, with an interval of 14 days between harvests. A representative subsample (100 fruits) was taken from each homogenized batch, and maturity index (MI) was determined based on skin color and pulp [39]. MIs are presented in Table 1.

Olive oil samples were obtained from olive fruits processed within 24 h after harvesting by a laboratory oil mill (Abencor, MC2 Ingenieria y Sistemas, Sevilla, Spain) equipped with a hammer crusher, vertical thermostated olive paste mixers, and a centrifuge. After milling olive fruits on the hammer crusher, kneading of the olive paste was performed for 35 min at 26 ± 2 °C. This was followed by a vertical centrifugation at 1370× *g* for 70 s and, at the end of processing, the oily must was collected and left to decant. All parts of the laboratory oil mill were washed between each olive fruit batch. Obtained oil samples were stored in dark brown glass bottles without headspace at 16–18 °C. All analyses were performed in triplicate.

### 2.3. Analyses of Olive Oil Quality Parameters

The market quality parameters of oils samples were assessed according to EU regulations [40]. Results of free fatty acids, peroxide value, spectrophotometric indices and panel test scores (as median of olive fruity note and median of sensory defects of all tested oils) of both varieties, and irrespective of the harvest period, classified samples into commercial grade “extra virgin olive oil” (Tables S1 and S2). Cultivar had no effect on the basic quality parameters of VOOs, which was expected given the nature of the parameters. Moreover, although analysis of variance determined the effect of harvest period on free fatty acid (FFA), peroxide value (PV) and K numbers (except K_270_ in Oblica VOOs), a clear correlation with the MI of the fruits was not established. Nevertheless, the oils produced from fruits of different MIs and processed immediately after harvest were not exposed to major hydrolytic and oxidative changes (Tables S1 and S2).

### 2.4. Analyses of Phenols

Total phenolic content (TPC) was determined following a slightly modified colorimetric Folin–Ciocalteu method [41]. Olive oil (10 g) was dissolved in an *n*-hexane (20 mL) and phenols were extracted with a water/methanol mixture (60:40, *w*/*w*) (50 mL). The extraction of phenols was repeated 2 more times and obtained methanol extracts were collected and combined. To remove oil residues, *n*-hexane (30 mL) was used, followed by evaporation of the extract to dryness on a rotary vacuum evaporator (Devarot, Elektromedicina, Slovenia). The dry extract was redissolved with methanol and 0.1 mL of extract was mixed with 5 mL of distilled deionized (dd) water and 0.5 mL of Folin–Ciocalteu reagent (Sigma-Aldrich, St Louis, MO, USA). After 5 min, 1 mL of saturated sodium carbonate solution was added, and the solution was diluted to 10 mL with dd H_2_O. After being kept in the dark for 60 min at room temperature, the absorbance was measured at 725 nm using a Cary 50 UV-VIS spectrophotometer (Varian, Palo Alto, CA, USA) versus prepared blank. The results were expressed as mg Gallic acid per kg of oil.

### 2.5. Analyses of Fatty Acid Composition 

Evaluation of fatty acid methyl ester was performed using an Agilent 6890N GC System (Santa Clara, CA, USA) equipped with a flame ionization detector (FID), prepared according to the ISO method (ISO 12966-2, 2011). Oil samples were subjected to alkaline treatment with 2,2,4-trimethylpentane (4 mL) and methanolic potassium hydroxide solution (0.2 mL). After vigorously shaking for 30 s, mixture was left to stratify and 2 phases were formed. The organic layer was separated and used for analysis. Chromatographic separation was performed on a DB-WAX column (30 m × 0.25 mm × 0.25 µm) with cyanopropyl-silicone as stationary phase (Agilent). For determination of fatty acid composition (ISO 5508, 1990), a flow rate of 1.5 mL/min of helium as a carrier gas was used. The operating conditions were the following: injector and detector temperatures were set at 250 °C and 280 °C, respectively; the starting temperature of the instrument oven was 60 °C and it was programmed to raise in intervals of 7 °C/min to a final temperature of 220 °C; final temperature was maintained for 17 min; split ratio was set to 30:1. The fatty acids in the olive samples were identified by comparison with the retention time of the standard mixture of fatty acids methyl esters. Calculation of the quantitative composition of fatty acids was carried out by the means of normalization surface method. The results were expressed as the percentage of total fatty acids present in olive oil. The percentages of total saturated (SFA), monounsaturated (MUFA) and polyunsaturated (PUFA) fatty acids were calculated, as well as their mutual ratios.

### 2.6. Quantitative Descriptive Analysis of Virgin Olive Oils

To establish the sensory profile of VOOs, quantitative descriptive analysis (QDA) was performed by a trained analytical taste panel consisting of eight experts in VOO tasting. The oil samples were evaluated according to the International Olive Council [42] methodology, using a modified profile sheet expanded with positive odor and taste descriptors (green fruity, leaves, grass, apple, almond, and bitter, pungent, sweet and astringent) [36] in order to attain a wide-ranging description of the oils’ organoleptic traits. Overall quality score (OQS) of olive oil, a scale from 1 (the lowest quality) to 9 (the highest quality), was applied according to the method described in the European Communities Regulation (1991). Olive oils rated with a score equal to or higher than 6.5 were classified in the category of extra virgin olive oils. The range of sensory analyst ratings was allowed at 0.25. Randomly coded and heated olive oil samples (15 g) to a temperature of 28 ± 2 °C were presented to the evaluators in blue glasses. 

### 2.7. Statistical Analysis 

The obtained data were analyzed using the Statistica software version 12.0 (StatSoft, Inc., Tulsa, OK, USA, 2013). Applied factorial ANOVA showed that the cultivars significantly differed in 20 out of 31 examined traits. Therefore, the data of each cultivar were processed independently. In order to determine the influence of crop year, growing site and harvest period on the measured parameters in the olive oil samples, a three-way analysis of variance (ANOVA) was applied. When *F*-tests were significant, the means were compared using Tukey’s honestly significant difference test at 5% significance level. Pearson linear correlation was used to relate the parameter investigated in the study to the climatic conditions during the year. At the end, multivariate analysis of the data that allows the identification of behavior patterns, and defines which variables differentiate between two or more a priori defined sets (PCA method), was run. Besides the data presented in this paper (basic quality parameters, total phenolic content, fatty acid composition, and sensory profile), concentration of tocopherol content (α, γ-tocopherol and total tocopherols) presented in Špika et al. [43] was used for obtaining the selected two-factor PCA model.

## 3. Results and Discussion 

### 3.1. Influence of Crop Year, Environmental Conditions of Two Olive Experimental Sites and Olive Cultivar on Virgin Olive Oil Chemical Composition and Sensory Characteristics

#### 3.1.1. Phenolic Content

**Figure 2:**
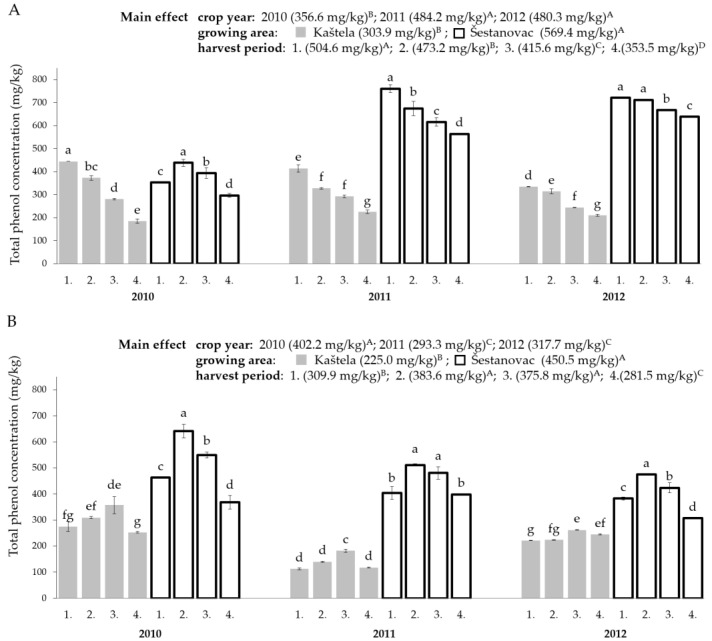
Total phenol concentration (mg/kg) of Oblica (**A**) and Leccino (**B**) virgin olive oils during ripening, obtained from two distinct olive orchards (Kaštela and Šestanovac) in three successive crop years. Bars labelled by different lowercase letters, for each crop year, are significantly different, while different uppercase letters indicate differences within main effects (crop year, growing site and harvest period), obtained by three-way ANOVA (Tukey’s test, *p* ≤ 0.05). 1–4, harvest period, see also Table 1.

Total phenolic content differed significantly depending on the crop year and studied cultivar (Figure 2). Namely, in Oblica VOOs, the highest average TPC was recorded in 2011 (year with the highest average daily air temperatures and lowest rainfall) and the lowest in 2010, the highest rainfall year (1397.9 mm, 1961.9 mm; Kaštela and Šestanovac respectively) with the lowest average daily air temperatures. The opposite was observed in Leccino VOOs. Most studies dealing with the effects of climate conditions on phenolic compounds suggest that water availability is an essential parameter for phenol synthesis [16,44,45,46,47]. The period during which amounts of water can influence the increase or decrease of phenols is the time from pit hardening to the stage of full fruit development [48], corresponding to the period from July to October in this research. 

In Table 2 correlation factors of TPC with climatic parameters (Figure 1) are presented, where a negative correlation with precipitation in September was observed. Water deficit can lead to increased synthesis of phenolic compounds in olive fruits and associated VOOs, since stress conditions can affect the activity of enzymes, primarily L-phenylalanine ammonium lyase (PAL), i.e., an enzyme responsible for the synthesis of phenolic compounds in olive fruit [49]. On the other hand, Dabbou et al. [50] reported the highest phenolic content in VOOs obtained from irrigated Arbequina olives. In summary, different cultivars adapt differently to climatic conditions, and differences in phenolic content and PAL activity may be related to the agronomic characteristics of each cultivar [50,51]; this is also visible in the behavior of the examined Oblica and Leccino cultivars (Figure 2). The noticed reverse relationship of rainfall and TPC during ripening (September) affected fruit dry matter content [52], decreased the amount of vegetable water and, thus, reduced the loss of phenols in the aqueous phase during processing. This diverse response of the tested olive cultivars to water availability provides an opportunity to examine, in the future, physiological differences of their responses to water supply that may have arisen during the selection of two cultivars in two different environments (Dalmatia and Tuscany).

Regarding the TPC of mononocultivar VOOs, growing site induced higher variability than crop year (*F* = 1168.4 and *F* = 9917.5; crop year and growing site, respectively, for Oblica) (*F* = 405.3 and *F* = 4734.8; crop year and growing site, respectively, for Leccino). As evident from Figure 2, oils of both studied cultivars had almost twice as high phenolic content at the location of higher altitude and lower mean daily temperatures, which was also confirmed with recorded negative correlation of TPC and temperature (Table 2). This is contrary to the earlier finding that warmer growing areas and lower altitudes boost the accumulation of larger amounts of phenolic substances in oils [13,17,53]. Higher phenol content was observed by Issaoui et al. [14] in VOOs obtained from higher altitudes, while Aguilera et al. [29] and Rotondi et al. [54] found different responses of cultivars tested with respect to growing site as significantly different in altitudes and climatic conditions. This confirms the hypothesis stated earlier that the content of phenolic compounds is related to the agronomic characteristics of each cultivar and the ability of the cultivar to adapt to different agro-climatic conditions.

Cultivars Oblica and Leccino differed significantly in TPC. In our study in Oblica, oils produced at laboratory scale TPC values varied from 185.0 to 760.3 mg/kg (average 436.7 mg/kg) while, for Leccino oils, values varied from 118.9 to 641.76 mg/kg (average 337.74 mg/kg) (Figure 2). These results are in line with studies showing different phenolic content in monocultivar oils that ranges from 40 to more than 4000 mg/kg [17,29,55,56,57].

#### 3.1.2. Fatty Acid Composition

Crop year had a significant influence on most fatty acids in both Oblica and Leccino VOOs (exceptions: heptadecenic, behenic and lignoceric in Oblica and lignoceric fatty acids in the Leccino) (Table 3 and Table 4, Tables S3 and S4). The average proportions of palmitic, linoleic and linolenic fatty acids were significantly lower, while the average proportion of oleic fatty acids was significantly higher in Oblica and Leccino VOOs from the year 2010 (the highest rainfall year with lowest average daily air temperatures) compared to the other two years studied. According to the correlation coefficients (Table 5), the fatty acids of the analyzed samples showed dependence on climatic conditions, which is consistent with the literature findings that lipid biosynthesis is influenced by environmental factors such as light, temperature and the amount of available water. Oleic fatty acid was negatively correlated, while palmitic, linoleic and linolenic fatty acids were positively correlated with the mean daily air temperatures at the time of intense growth and ripening of the olive fruits (Table 5). During the same period, lower mean daily temperatures were recorded in 2010 (Figure 1) and, in the same year, higher average oleic fatty acids and significantly lower average palmitic, linoleic and linolenic fatty acids were observed in VOOs of both varieties (Table 3 and Table 4). García-Inza et al. [32] noticed the same behavior in a study on the influence of temperature on fatty acid composition. Although the temperature of the growing area is a parameter that has a larger impact, precipitation can also affect de novo fatty acid biosynthesis that takes place in plastids. Extension of palmitoyl-ACP to stearoyl-ACP occurs by the condensation enzyme *β*-ketoacyl-ACP synthetase II (KAS II). This step determines the C16/C18 ratio and directly affects the degree of unsaturation of the oil [58]. Oleic fatty acid content was negatively correlated, while linoleic fatty acid content was positively correlated with precipitation in July (Table 5). In that period in 2011, significantly higher rainfall was recorded at both locations (Kaštela; 21.8 mm, 130.8 mm, 8.8 mm; 2010, 2011 and 2012, respectively) (Šestanovac; 13.2 mm, 179.6 mm, 9.0 mm; 2010, 2011 and 2012, respectively) (Figure 1), which may have influenced the decreased activity of KAS II [59,60]. As hypothesized by Caruso et al. [61], irrigation had more significant effect in warmer years and/or warmer areas, which was shown to be strongly cultivar dependent. Moreover, irrigation during fruit development increased the content of linoleic fatty acid and decreased the content of oleic and palmitoleic fatty acids [61,62]. Consistent results were recorded in the year 2011 of this study (Table 3 and Table 4). It is evident that crop years abundant with rainfall and higher temperatures affected the biosynthesis of fatty acids in Oblica and Leccino VOOs, their interactions and ratios, and, thus, final product quality and stability.

Oils obtained from the two experimental sites significantly differed in content of the majority of fatty acids in Oblica (exception: heptadecenic, stearic, behenic and lignoceric fatty acids) (Table 2 and Table S2) and Leccino (except behenic fatty acid) (Table 3 and Table S3). The content of palmitic fatty acid was significantly higher in Kaštela (Table 2 and Table 3), and stearic acid followed the same trend. Meanwhile, a significant difference between growing sites in Oblica VOOs was not observed. In general, a higher content of saturated fatty acids was recorded in VOOs of Oblica and Leccino from Kaštela (Supplemental Tables S3 and S4). In both monocultivar oils from Šestanovac (higher altitude), a higher oleic fatty acid content was observed compared to VOOs from Kaštela. The oils obtained at Šestanovac of both studied cultivars had, in turn, lower polyunsaturated fatty acids (PUFA) (Table 2 and Table 3, Tables S3 and S4), as was earlier published for oils of higher altitudes [29]. Still, results that demonstrate opposite cultivar behavior have been published. Rotondi et al. [54] stated higher PUFA in oils of higher altitudes for the Leccio del Corno VOOs, while, for the Leccino oils in the same study, growing site did not affect their content. 

Fatty acid biosynthesis was influenced by climatic parameters in the present study; temperature and rainfall parameters differed in two monitored olive sub-regions (Table 5, Figure 1). Since Šestanovac is an orchard that has lower mean daily temperatures, accordingly, higher stearic, linoleic and linolenic fatty acids and a lower oleic and monounsaturated fatty acids content were observed in Oblica and Leccino VOOs from this growing site (Table 3 and Table 4). Conditionality of VOO fatty acid composition with climatic parameters, confirmed by the correlation factors (Table 5), is compliant with “Ivanov rule”: “The proportion of linoleic acid increases with decreasing temperature, in contrast to the proportion of oleic acid” [63]. Higher values of 18:1/18:2 ratios were recorded in Šestanovac VOOs (Table 3 and Table 4), and the same trend is visible in MUFA/PUFA ratios (Tables S3 and S4).

According to fatty acid composition, samples of both cultivars were classified as extra virgin olive oil [25]. Cultivars significantly differed in the content of fatty acids (except heptadecenic, linolenic and behenic fatty acids), which is in line with the literature findings, according to which fatty acid composition is used for the characterization and evaluation of VOOs [29]. Based on the individual content of main and dominant fatty acids (oleic, palmitic and linoleic fatty acids), Uceda [64] classified VOOs into five categories: very low, low, medium, high or very high content of a single fatty acid. Accordingly, Oblica had a mean oleic fatty acid content ranging from 64.8% to 76.1%, and averaged 70.4% of high palmitic fatty acid content (average 13.43%, ranged from 10.12% to 15.31%), as well as a medium content of linoleic fatty acid (average 11.22%). Mean content of oleic fatty acid (average 72.9%) and very high palmitic fatty acid content (average 14.96%) characterized Leccino VOOs. Polyunsaturated linoleic fatty acid content was of an average of 6.99% (5.53–9.74%), and defined Leccino as VOOs with its low content [64]. Fatty acid contents are in line with previous studies on Oblica and Leccino [54,55,65]. The C18:1/C18:2 ratio of Oblica VOOs was shown to be lower compared to Leccino VOOs (Table 3 and Table 4). The same trend is evident in the MUFA/PUFA ratio. The resulting C18:1/C18:2 ratio can be considered stable; although, compared with Picual VOOs (C18:1/C18:2–26) [60], high care must be taken to prevent oxidative changes.

#### 3.1.3. Sensory Profile

**Figure 3:**
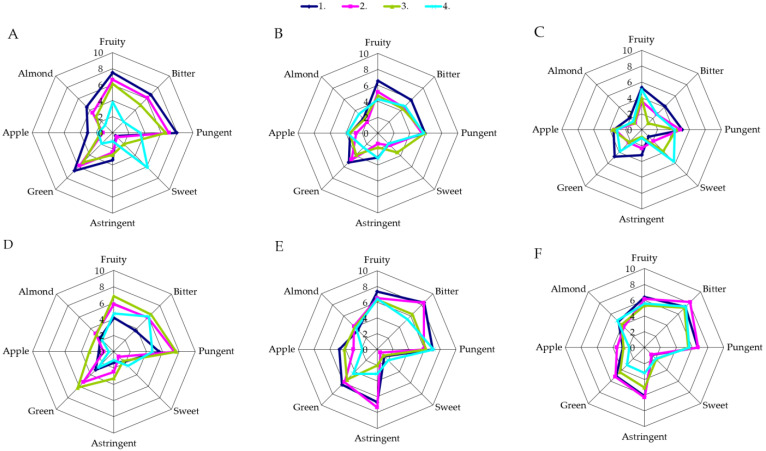
Sensory profile of Oblica virgin olive oils obtained in 3 successive crop years from the 2 distinct olive orchards at 4 harvest periods. (**A**)—Kaštela 2010, (**B**)—Kaštela 2011, (**C**)—Kaštela 2012; (**D**)—Šetanovac 2010, (**E**)—Šetanovac, 2011, (**F**)—Šetanovac 2012; 4 harvest periods—line colors 1–4 within each graph.

**Figure 4:**
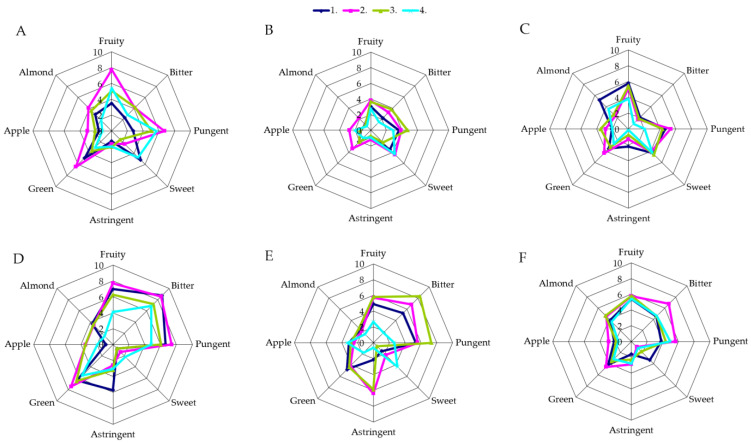
Sensory profile of Leccino virgin olive oils obtained in 3 successive crop years from the 2 distinct olive orchards at 4 harvest periods. (**A**)—Kaštela 2010, (**B**)—Kaštela 2011, (**C**)—Kaštela 2012; (**D**)—Šetanovac 2010, (**E**)—Šetanovac, 2011, (**F**)—Šetanovac 2012; 4 harvest periods—line colors 1–4 within each graph.

The results of quantitative descriptive analysis indicate that the sensory profile of single varietal oil was significantly altered among particular crop years (Figure 3 and Figure 4, Tables S3 and S4). Precisely, crop year induced the highest variability (F-statistic value) for attributes astringent and apple in Oblica and bitter and green in Leccino VOOs. A higher average overall quality score was observed in the year 2010 for both monocultivar oils (statistical significance was observed only for Oblica) (Figure S1). 

In the same year, (if compared with the warmest year—2012), Leccino VOOs had a more pronounced pungent sensation, while the oils obtained in year 2012 showed the lowest intensity of the bitter; consequently, sweet taste was more pronounced. The trend of changes in Oblica VOOs was similar but statistically not confirmed (Table S5). The correlation coefficients of the sensory properties with the climatic parameters in the period under consideration (Figure 1) are shown in Table 6. The taste properties bitter and pungent correlate negatively with the mean daily temperatures and with rainfall from September to November. Since taste sensory properties depend mostly on the concentration of phenolic substances [66], correlations of taste properties with climatic parameters follow the same trend as phenolic substances.

The sensory profiles of VOOs from the two cultivars studied varied significantly with respect to growing site (Figure 3 and Figure 4, Tables S5 and S6). There was no difference in the intensities of the apple attribute in Oblica and the OQS of oils of both cultivars (Tables S5 and S6, Figure S1). Differences, but without uniform outcome, in sensory profiles with respect to growing area were reported in previous studies. Higher fruitiness, bitterness and pungency were observed in Chemlali oils from higher altitude areas compared to oils from southern lower altitude areas [14].

Leccino oils from considerably different locations, Italy and Spain, have been described as oils of similar sensory attributes with slight differences [29,54]. The results discussed in this paper (Figure 3 and Figure 4, Tables S5 and S6) indicate significantly different sensory profile of both VOOs with respect to site of cultivation, primarily in the intensity of the taste attribute bitter. Namely, oils from the cv. Oblica, obtained from fruits harvested at Kaštela, can be described as harmonious oils with medium intensity of fruitiness, clearly expressed green tones (grass, green fruits and vegetables) and scarce aromas of apple and almond. The sweetness of the taste was of mild intensity while bitter and pungent were medium expressed (Figure 3A−C, Table S5). Intense bitterness and pungency and mild astringency were noted in the oils from Šestanovac. The green flavor was significantly more pronounced compared to VOOs from Kaštela, as well as fruity, while sweet is hardly noticeable (Figure 3D−F, Table S5). Leccino VOOs from Kaštela were harmonious oils with low intensity of bitter and medium intensities of pungent and fruity. These oils had a mild aroma of green, almond and apple, with a medium sweet taste attribute (Figure 4A−C, Table S6). The sensory profile of Leccino VOOs from Šestanovac was of medium pronounced fruitiness, a slightly more pronounced pungency and intense bitterness. The low intensity of astringency was noted in the mouth but lingered for a long time (Figure 4D–F, Table S6). 

The cultivars differed significantly in sensory properties as follows: bitter, pungent, sweet and astringent, and VOOs OQS (Figure 3 and Figure 4, Figure S1). On average, Oblica VOOs had higher overall quality scores than Leccino oils (Figure S1). Oils from Oblica can be described as harmonious medium-fruity oils with distinct green tones (herbs, green fruits and vegetables), and mild apple and almond scents. The taste exhibited a characteristic of slightly mild intensity of sweet, medium-noticeable bitterness and medium-to-intense pungency, accompanied by slightly pronounced astringency (Figure 3). Leccino oils had well-balanced bitter and sweet taste attributes, and were of medium intensity fruity, with a slight almond and a barely noticeable apple sensory characteristic. Mild-to-medium *bitter* and almost equal pronounced pungent were main taste traits (Figure 4). 

### 3.2. Influence of the Olive Maturity Index on Virgin Olive Oils Chemical Composition and Sensory Characteristics

The olive maturity index had a significant effect on the content of polyphenols in VOOs of Oblica and Leccino (Figure 2A,B). For Oblica, mostly reduction by the ripening was noted (except year 2010 at the Šestanovac). A decrease in phenols throughout the ripening period was also observed in Spanish cultivars Arbequina, Picual and Cornicabra [67]. Oblica VOOs from Kaštela decreased by almost 50%, on average, during ripening (Figure 2A). The loss of TPC in Oblica VOOs from Šestnovac was less expressed (approximately 18% compared to the initial value) and it covered the entire period of ripening under observation. The phenols in Leccino oils from both sites increased by ripening to a certain point when the maximum was reached, followed by an average decrease of 25% (Figure 2B). In the three years of research, Leccino VOOs from Kaštela achieved the maximum TPC at the third harvest (mid-October, Figure 2B), in which the olives’ maturity index was from 3.5 to 4.0 (Table 1), whereas the maximum TPC in oils from Šestanovac was recorded in fruit harvested at the beginning of October (second harvest), with the lower MI of olive fruits (MI from 1.72 to 3.3). Baccouri et al. [7] recorded the same pattern of changes in VOOs from Chetoui and Chemlali.

Analysis of variance revealed that the MI of olive fruits had a significant effect on the majority of the fatty acids identified (Table 3 and Table 4, Tables S3 and S4). During ripening, a decrease in palmitic fatty acid content was observed in the oils of both varieties and of both growing sites. This corresponds with the results described for most olive cultivars [60,68,69]. Cultivar-dependent, stearic acid showed dissimilar patterns during ripening; a constant decrease (Oblica 2012, Leccino 2010 and 2012), a slight increase during ripening (Oblica 2011) and no significant difference with MI increase (Oblica 2010) (Table 3 and Table 4). Salvador et al. [70] found an increase in stearic fatty acid in a study of oils from Cornicabra, as did Beltrán et al. [60] in the oils from Picual cultivar. On the contrary, a decrease in stearic acid was observed in Chemlali VOOs [7]. The irregular changes may be explained by the effect of dilution, i.e., the absolute amount of other fatty acids changes with ripening, which influences the changes in the constant content of stearic fatty acid [71]. With the fruit MI increases, the oleic fatty acid increased in all years in both varietal oils (Table 3 and Table 4) [60,72], although opposite behavior was noted [70,73]. Linolenic fatty acid decreased during ripening for both studied VOOs and at both growing sites. A significant decrease of C18:1/C18:2, with increasing fruit MI, was noted in Oblica (Table 3), while the opposite was recorded in Leccino VOOs (Table 4). Biochemical studies have shown that PUFAs are formed by desaturation of oleic fatty acid in olive fruits, as well as other plant species, by the action of desaturase [74,75]. The results of this research showed that the examined cultivars respond differently under the same pedological and climatic conditions, and this may be related to the hypothesis that cultivars have different enzymatic capacity for the desaturation of fatty acids [76]. Thus, the presented results indicate that timely fruit harvest can help achieve higher VOO stability and quality.

According to the results of three-way ANOVA, MI was the factor that significantly influenced sensory profiles of monocultivar oils produced at laboratory scale (Tables S5 and S6, Figure 3 and Figure 4). As the olives ripened, the intensity of the fruitiness decreased in Oblica VOOs. Comparing the first harvest (unripe fruits) with last harvest (overripe olives), the largest fruitiness loss was recorded in oils obtained from fruits harvested in Kaštela (year 2010) (intensity decline 7.5 to 3.8) (Figure 3A–C). On the other hand, ***fruitness*** of Leccino VOOs from both growing sites and for all three years of research followed a Gauss curve (Figure 3). Namely, volatile substances are responsible for the specific VOO sensory attributes released during the extraction of olives in the lipoxygenase pathway. Concentration of the C6 and C5 volatile substances decreases with ripening [77], as result of the enzymes’ activity reduction with ripening, and are highly correlated with fruitiness [37,78]. The development of these substances is associated with the content of PUFA, as they are a substrate of lipoxygenase, while phenolic substances are inhibitors of the same enzymes [79]. The most probable reason for lower fruitiness in the first harvest periods of Leccino (oils from unripe fruit) (Figure 4), lies in the different activity of the enzymes, the availability of the substrate and the presence of inhibitors. 

In all three years of research, along with ripening, a decrease in bitterness was recorded in Oblica VOOs from Kaštela (Figure 3). Although the decrease rates were specific for each crop year, the highest loss was recorded in 2010 (from 6.7 to 2.0). The same was noted for the taste attribute pungent. Oblica VOOs from Šestanovac had the highest intensities of bitter and pungent at MI ~ 1 (second and third harvests). Observed changes of bitter and pungent in Oblica and in Leccino (Figure 3 and Figure 4) were consistent with changes of TPC (Figure 2) and secoiridoids (data not shown). Such findings confirm the link between taste sensory attributes and phenolic compounds [66]. The highest values of sweetness were recorded in the oils obtained from overripe fruits for all three years of research (Figure 3 and Figure 4). This was expected, considering that this attribute is more prominent in the absence and weakening of bitterness and pungency. As opposed to sweet, a higher astringency sensation was noted only in Oblica VOOs from Šestanovac (Figure 4D–F). The astringent of medium intensity was characteristic mainly in the early period of ripening (green olives), and decreased with MI, resulting from the presence of phenols, flavonoids and 3,4-DHPEA-EDA in these samples [35]. 

With the MI increase, Oblica oils from Kaštela resulted in a decreased overall quality score (Figure S1A). The decline of OQS with ripening was recorded for the other cultivars [80]. The best rated VOOs of Oblica from Šestanovac were the oils obtained from fruits harvested during October, corresponding to MI from 0.25 to 2.94 (Figure S1). The variations of the OQS were a reflection of changes of the chemical composition of VOOs, their content of primary antioxidants and as a consequence of evaluated sensory properties. Generally, in Oblica from Kaštela, OQS decreased during the entire observed period, and the highest scores were recorded from early harvests where the MI were up to 0.79. In Leccino VOOs from both locations, a harmony of flavor and taste properties was mostly achieved in the second and third fruit harvest, which coincides with October and MI from 1.72 to 3.96 (Figure S1B).

### 3.3. The Principal Component Analysis Reveals the Central Role of Cultivar in the Virgin Olive Oils Chemical Composition and Sensory Characteristics 

**Figure 5:**
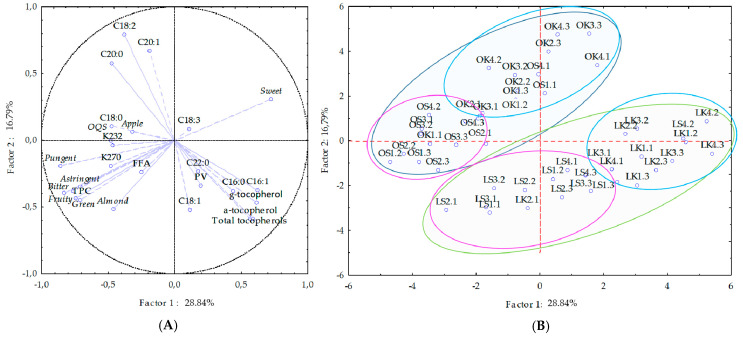
(**A**) Projection of the variables (basic quality parameters (FFA, PV, K numbers), fatty acid composition, tocopherol composition, total phenols (TPC) and sensory properties) of the first two factors, obtained by principal component analyses; and (**B**) projection of the virgin olive oil samples of Oblica (O) and Leccino (L) grown in Kaštela (K) and Šestanovac (S) during ripening (1–4, harvest period, also see Table 1) in 3 successive crop years (1–3, number after the point) in the two-dimensional space of first 2 factors.

In order to determine the main sources of variance, as well as the potential relationship between the analyzed parameters and the VOO samples, data collected over the three years were processed through PCA. Figure 5A shows the projection of the parameters included (basic quality parameters, fatty acid composition, tocopherol content, polyphenols and sensory profile). Palmitic and palmitoleic fatty acids correlate positively with Factor 1, while stearic fatty acids correlate negatively with the same factor. High positive correlation of Factor 1 was recorded with linoleic, gedoleic and behenic, while negative correlation with oleic fatty acid (Figure 5A). Thus, from the projection, it can be seen that oleic fatty acid content was inversely proportional to linoleic fatty acid content [30,60]. The α–, γ–, total tocopherol content and the taste attribute *sweet* were also positively correlated, while TPC and almost all properties of QDA of VOOs were highly negatively correlated with Factor 1. The dependence of phenolic compounds on VOO sensory properties was confirmed (Figure 5A).

VOOs from all three years of research were primarily separated by the cultivar (Figure 5B). The Oblica VOO samples were located mostly on the positive side of Factor 2 and were characterized by a higher content of linoleic, arachidonic and gadoleic fatty acids. They had more pronounced evaluated flavor and taste, and obtained a better sensory score. On the negative side of Factor 2 were Leccino VOOs, with higher levels of palmitic and oleic fatty acids and higher tocopherol content (Špika et al. 2016). The results of the analysis of the main components showed that the composition of fatty acids, tocopherols, phenolics and sensory profile are characteristic for a cultivar. Within those main groups, the samples were divided with regard to the growing site (with a few exceptions), and the differences were more clearly visible among Oblica samples. The crop year was not a factor that led to the grouping of samples accessed by PCA, but it still affected the biosynthesis of natural VOOs antioxidants, fatty acids, and their interactions and ratios, and, thus, final product quality and stability.

Preformed PCA showed nicely distinguished monocultivar oils by content of naturally presented antioxidants and sensory features, thus revealing the central role they play in discrimination among cultivars. 

## 4. Conclusions

Expansion of olive production from traditional areas is an opportunity to gain insights into likely crop responses in new or modified growing environments. The discussed results can be considered as valuable data in providing information on environmental conditions under which olive fruit growth affects the content of phenolic compounds, fatty acid composition and sensory traits. It was demonstrated that the studied parameters in VOOs depend primarily on cultivar, then growing site and crop year, whereas harvesting period is ranked as having the least impact. However, the response of the two cultivars to stated factors was not equal, indicating the need to take this into consideration when assessing desired oil quality. Although all the produced oils were of excellent quality, it was showed that there is large variability of phenols and sensory attributes within each monocultivar oil, which opens the possibility of meeting the increasingly demanding market of VOO. 

## Figures and Tables

**Table 1:** Fruit maturity index of Oblica and Leccino cultivars grown in Kaštela and Šestanovac.

Crop Year	Harvest Period	Oblica		Leccino	
		Kaštela	Šestanovac	Kaštela	Šestanovac
2010	1.	0.00	0.00	2.36	1.83
	2.	0.56	0.25	3.67	3.30
	3.	1.12	1.05	3.90	3.82
	4.	2.73	3.97	4.12	4.85
2011	1.	0.00	0.00	2.49	1.41
	2.	0.48	0.44	3.50	2.01
	3.	1.70	2.94	4.00	3.96
	4.	3.94	3.87	4.05	4.10
2012	1.	0.37	0.29	2.11	1.05
	2.	0.79	0.56	2.31	1.72
	3.	2.12	1.27	3.15	2.56
	4.	2.86	2.84	3.48	3.37

**Table 2:** Correlation factors of virgin olive oil phenol concentration and microclimate parameters (rainfall and mean temperature; Figure 1) in the period of olives’ intensive growth and ripening.

	Rainfall					Mean Temperature				
	Jul	Aug	Sep	Oct	Nov	Jul	Aug	Sep	Oct	Nov
TPC ^1^	ns ^2^	ns	−0.306	−0.202	0.343	−0.284	−0.413	−0.270	−0.266	−0.577

^1^ TPC—total phenol concentration (mg/kg); ^2^ statistically significant difference at *p* ≤ 0.05, ns—correlation between parameters not significant *p* ≤ 0.05.

**Table 3:** Fatty acid composition of Oblica virgin olive oils during ripening obtained from 2 distinct olive orchards (Kaštela and Šestanovac) in 3 successive crop years.

Factor			Fatty Acid							
			C16:0	C16:1	C18:0	C18:1	C18:2	C18:3	C18:1/C18:2	MUFA/SFA
**2010**	**Kaštela**	1.	14.23 ± 0.03a	0.81 ± 0.01a	2.11 ± 0.01ab	71.15 ± 0.03g	9.71 ± 0.02e	0.81 ± 0.01b	7.33 ± 0.01e	4.24 ± 0.01h
		2.	13.71 ± 0.01b	0.81 ± 0.01a	2.15 ± 0.13a	71.13 ± 0.04g	10.21 ± 0.02b	0.81 ± 0.01b	6.97 ± 0.01g	4.36 ± 0.04g
		3.	12.92 ± 0.02c	0.81 ± 0.01a	2.11 ± 0.01ab	72.23 ± 0.03f	10.12 ± 0.03c	0.71 ± 0.01c	7.15 ± 0.03f	4.68 ± 0.01f
		4.	12.14 ± 0.02e	0.71 ± 0.01b	2.01 ± 0.01bc	72.92 ± 0.02e	10.43 ± 0.02a	0.71 ± 0.01c	7.00 ± 0.01g	5.03 ± 0.01c
	**Šestanovac**	1.	12.93 ± 0.02c	0.71 ± 0.01b	2.02 ± 0.03abc	73.92 ± 0.02c	8.33 ± 0.03g	0.91 ± 0.01a	8.89 ± 0.03a	4.78 ± 0.01e
		2.	12.52 ± 0.02d	0.61 ± 0.01c	2.02 ± 0.03abc	73.53 ± 0.04d	9.41 ± 0.01f	0.81 ± 0.01b	7.82 ± 0.01b	4.91 ± 0.01d
		3.	10.82 ± 0.05f	0.51 ± 0.01d	2.03 ± 0.04abc	75.03 ± 0.03b	9.71 ± 0.01e	0.71 ± 0.01c	7.74 ± 0.01c	5.62 ± 0.02b
		4.	10.12 ± 0.03g	0.51 ± 0.01d	1.91 ± 0.01c	76.06 ± 0.05c	9.92 ± 0.03d	0.71 ± 0.01c	7.68 ± 0.02d	6.06 ± 0.01a
**2011**	**Kaštela**	1.	14.81 ± 0.03b	1.11 ± 0.01b	2.01 ± 0.01d	66.01 ± 0.01f	14.10 ± 0.02d	1.01 ± 0.01a	4.69 ± 0.01f	3.88 ± 0.01f
		2.	15.22 ± 0.02a	1.41 ± 0.01a	2.11 ± 0.01c	64.81 ± 0.01h	14.41 ± 0.01a	0.91 ± 0.01b	4.51 ± 0.01h	3.70 ± 0.01h
		3.	14.61 ± 0.02c	1.01 ± 0.01c	2.31 ± 0.01a	65.81 ± 0.01g	14.31 ± 0.01b	0.91 ± 0.01b	4.61 ± 0.01g	3.82 ± 0.01g
		4.	13.51 ± 0.01f	0.81 ± 0.01e	2.31 ± 0.01a	67.12 ± 0.03e	14.21 ± 0.01c	0.81 ± 0.01c	4.73 ± 0.01e	4.18 ± 0.01d
	**Šestanovac**	1.	14.41 ± 0.02d	0.91 ± 0.01d	2.21 ± 0.01b	69.31 ± 0.01d	11.11 ± 0.01f	0.91 ± 0.01b	6.25 ± 0.01c	4.11 ± 0.01e
		2.	13.71 ± 0.01e	0.91 ± 0.01d	2.21 ± 0.01b	70.21 ± 0.01c	11.11 ± 0.01f	0.81 ± 0.01c	6.33 ± 0.01b	4.34 ± 0.01c
		3.	13.11 ± 0.02g	0.81 ± 0.01e	2.11 ± 0.01c	71.11 ± 0.01b	11.11 ± 0.01f	0.71 ± 0.01d	6.41 ± 0.01a	4.58 ± 0.01b
		4.	12.31 ± 0.01h	0.71 ± 0.01f	2.31 ± 0.01a	71.51 ± 0.01a	11.51 ± 0.02e	0.71 ± 0.01d	6.22 ± 0.01d	4.78 ± 0.01a
**2012**	**Kaštela**	1.	15.31 ± 0.06a	1.11 ± 0.01b	2.21 ± 0.01b	67.51 ± 0.05f	11.82 ± 0.04d	0.91 ± 0.01a	5.72 ± 0.03d	3.80 ± 0.02f
		2.	14.91 ± 0.03b	1.02 ± 0.03c	2.21 ± 0.01b	67.72 ± 0.04f	12.21 ± 0.03c	0.81 ± 0.01b	5.55 ± 0.01e	3.89 ± 0.01f
		3.	14.41 ± 0.09c	1.00 ± 0.01c	2.11 ± 0.04c	67.91 ± 0.01e	13.61 ± 0.12a	0.91 ± 0.01a	5.00 ± 0.05f	4.04 ± 0.02e
		4.	13.71 ± 0.01d	1.01 ± 0.01c	2.11 ± 0.01c	67.81 ± 0.02f	13.31 ± 0.03b	0.81 ± 0.02b	5.10 ± 0.01f	4.18 ± 0.01d
	**Šestanovac**	1.	14.51 ± 0.02c	1.21 ± 0.03a	2.40 ± 0.07a	69.80 ± 0.05d	10.11 ± 0.01e	0.91 ± 0.01a	6.91 ± 0.01c	4.04 ± 0.01e
		2.	13.81 ± 0.06d	1.11 ± 0.01b	2.22 ± 0.03b	71.31 ± 0.10c	9.71 ± 0.04f	0.81 ± 0.01b	7.36 ± 0.02b	4.39 ± 0.02c
		3.	12.82 ± 0.04e	1.01 ± 0.01c	2.21 ± 0.01b	72.41 ± 0.07b	9.81 ± 0.01f	0.71 ± 0.01c	7.39 ± 0.01b	4.73 ± 0.01b
		4.	11.91 ± 0.02f	1.01 ± 0.01c	2.22 ± 0.02b	74.03 ± 0.04a	9.21 ± 0.04g	0.71 ± 0.01c	8.04 ± 0.03a	5.13 ± 0.01a
**Year**	2010		12.42 ± 1.33c	0.68 ± 0.13c	2.04 ± 0.09b	73.24 ± 1.69a	9.73 ± 0.63c	0.77 ± 0.08c	7.57 ± 0.60a	4.96 ± 0.59a
	2011		13.96 ± 0.94a	0.96 ± 0.22b	2.19 ± 0.11a	68.23 ± 2.50c	12.73 ± 1.57a	0.84 ± 0.11a	5.47 ± 0.86c	4.17 ± 0.37c
	2012		13.92 ± 1.08b	1.06 ± 0.08a	2.21 ± 0.09a	69.81 ± 2.40b	11.22 ± 1.66b	0.82 ± 0.08b	6.38 ± 1.13b	4.27 ± 0.44b
	*F*		21,304	14,436.6	205.1	148,383	64,983	5703	1018	20,007
	*p*		***	***	***	***	***	***	***	***
**Growing site**										
	Kaštela		14.12 ± 0.94a	0.97 ± 0.19a	2.14 ± 0.10	68.51 ± 2.60b	12.37 ± 1.80a	0.84 ± 0.09a	5.70 ± 1.08b	4.15 ± 0.39b
	Šestanovac		12.75 ± 1.3b	0.83 ± 0.23b	2.15 ± 0.14	72.35 ± 2.10a	10.09 ± 0.92b	0.78 ± 0.09b	7.25 ± 0.82a	4.79 ± 0.58a
	*F*		39,233	5169.3	1.6	250,172	112,897	10,009	124,865	42,694
	*p*		***	***	*ns*	***	***	***	***	***
**Harvest period**										
	1.		14.37 ± 0.76a	0.97 ± 0.19a	2.16 ± 0.14	69.62 ± 2.61d	10.86 ± 1.87c	0.91 ± 0.07a	6.63 ± 1.36a	4.14 ± 0.33d
	2.		13.98 ± 0.92b	0.97 ± 0.26a	2.15 ± 0.09	69.78 ± 2.89c	11.18 ± 1.77b	0.82 ± 0.04b	6.42 ± 1.16c	4.26 ± 0.40c
	3.		13.12 ± 1.29c	0.86 ± 0.19b	2.14 ± 0.10	70.75 ± 3.15b	11.44 ± 1.91a	0.77 ± 0.10c	6.38 ± 1.23d	4.58 ± 0.60b
	4.		12.28 ± 1.22d	0.79 ± 0.19c	2.14 ± 0.16	71.57 ± 3.31a	11.43 ± 1.86a	0.74 ± 0.05d	6.46 ± 1.28b	4.89 ± 0.67a
	*F*		17,943	2383.2	1.0	14,073	1622	15,333	203,681	71,255
	*p*		***	***	*ns*	***	***	***	***	***

Means marked by different lowercase letters (a–h) in column (for each crop year) and for each main factor (crop year, growing site and harvest period) are significantly different (Tukey’s test, *p* ≤ 0.05). Significance: ***—*p* ≤ 0.001, ns—not significant. Harvest period, 1–4, also see Table 1. Values were calculated as the percentage of the total.

**Table 4:** Fatty acid composition of Leccino virgin olive oils during ripening, obtained from 2 distinct olive orchards (Kaštela and Šestanovac) in 3 successive crop years.

Factor			Fatty Acid							
			C16:0	C16:1	C18:0	C18:1	C18:2	C18:3	C18:1/C18:2	MUFA/SFA
**2010**	**Kaštela**	1.	15.07 ± 0.04a	0.91 ± 0.01d	2.61 ± 0.01a	71.73 ± 0.05h	7.92 ± 0.02a	0.91 ± 0.01a	9.07 ± 0.02g	3.98 ± 0.01f
		2.	14.91 ± 0.01b	1.11 ± 0.01c	2.31 ± 0.01b	72.71 ± 0.02g	7.11 ± 0.01b	0.81 ± 0.01b	10.25 ± 0.01f	4.15 ± 0.01e
		3.	14.51 ± 0.01c	1.41 ± 0.01a	2.11 ± 0.01d	73.92 ± 0.03e	6.61 ± 0.01c	0.71 ± 0.01c	11.20 ± 0.01e	4.43 ± 0.01d
		4.	14.31 ± 0.01e	1.41 ± 0.01a	1.91 ± 0.01f	74.84 ± 0.06c	6.02 ± 0.02e	0.61 ± 0.01d	12.45 ± 0.03c	4.62 ± 0.01c
	**Šestanovac**	1.	15.12 ± 0.02a	0.81 ± 0.01e	2.31 ± 0.01b	73.12 ± 0.02f	7.11 ± 0.02b	0.81 ± 0.01b	10.29 ± 0.02f	4.15 ± 0.01e
		2.	14.41 ± 0.01d	1.05 ± 0.05d	2.21 ± 0.01c	74.71 ± 0.02d	6.12 ± 0.02d	0.71 ± 0.01c	12.23 ± 0.04d	4.43 ± 0.01d
		3.	14.01 ± 0.01g	1.11 ± 0.01c	2.02 ± 0.03e	75.82 ± 0.03a	5.61 ± 0.01f	0.61 ± 0.01d	13.54 ± 0.01b	4.65 ± 0.01b
		4.	13.21 ± 0.01f	1.21 ± 0.01b	2.21 ± 0.01c	75.61 ± 0.01b	5.53 ± 0.04g	0.61 ± 0.01d	13.68 ± 0.08a	4.88 ± 0.01a
**2011**	**Kaštela**	1.	15.21 ± 0.02c	1.21 ± 0.01d	1.71 ± 0.01d	71.75 ± 0.01e	8.45 ± 0.05d	0.91 ± 0.01a	8.50 ± 0.05e	4.22 ± 0.01e
		2.	15.41 ± 0.03b	1.21 ± 0.01d	1.71 ± 0.01d	71.31 ± 0.01f	9.13 ± 0.01c	0.91 ± 0.01a	7.82 ± 0.01f	4.15 ± 0.01f
		3.	14.72 ± 0.02e	1.21 ± 0.01d	1.81 ± 0.01c	70.21 ± 0.01g	9.44 ± 0.05b	0.91 ± 0.01a	7.45 ± 0.04g	4.23 ± 0.01e
		4.	14.12 ± 0.02g	1.31 ± 0.01c	1.91 ± 0.01b	71.33 ± 0.03f	9.74 ± 0.02a	0.81 ± 0.01b	7.33 ± 0.02h	4.42 ± 0.01c
	**Šestanovac**	1.	15.51 ± 0.01a	1.11 ± 0.01e	2.01 ± 0.02a	72.81 ± 0.02d	6.83 ± 0.03e	0.91 ± 0.01a	10.68 ± 0.04d	4.13 ± 0.01g
		2.	15.11 ± 0.02d	1.41 ± 0.01b	1.91 ± 0.02b	73.72 ± 0.03b	6.32 ± 0.02g	0.71 ± 0.01c	11.68 ± 0.03b	4.34 ± 0.01d
		3.	14.51 ± 0.01f	1.31 ± 0.02c	1.61 ± 0.01e	73.54 ± 0.01c	6.41 ± 0.01f	0.81 ± 0.01b	11.49 ± 0.02c	4.54 ± 0.01b
		4.	14.02 ± 0.02h	1.61 ± 0.01a	1.71 ± 0.01d	75.21 ± 0.01a	6.12 ± 0.01h	0.61 ± 0.01d	12.30 ± 0.01a	4.79 ± 0.01a
**2012**	**Kaštela**	1.	17.13 ± 0.21a	1.21 ± 0.01g	2.31 ± 0.01a	69.06 ± 0.07h	8.41 ± 0.02a	1.11 ± 0.01a	8.22 ± 0.02h	3.53 ± 0.04g
		2.	16.11 ± 0.09c	1.70 ± 0.06c	1.91 ± 0.01c	71.71 ± 0.02f	6.71 ± 0.01d	0.91 ± 0.03c	10.71 ± 0.01e	3.99 ± 0.01e
		3.	15.82 ± 0.03c	2.01 ± 0.02b	1.81 ± 0.01d	72.81 ± 0.04c	6.04 ± 0.06f	0.71 ± 0.01e	12.07 ± 0.13c	4.16 ± 0.01c
		4.	15.81 ± 0.01c	2.31 ± 0.01a	1.81 ± 0.01d	72.61 ± 0.01d	5.91 ± 0.04g	0.71 ± 0.01e	12.31 ± 0.09b	4.16 ± 0.01c
	**Šestanovac**	1.	16.61 ± 0.06b	1.21 ± 0.01g	2.11 ± 0.01b	70.50 ± 0.07g	7.41 ± 0.03b	1.01 ± 0.01b	9.53 ± 0.03g	3.74 ± 0.02f
		2.	15.70 ± 0.02c	1.41 ± 0.01f	1.81 ± 0.01d	72.12 ± 0.05e	7.11 ± 0.01c	0.91 ± 0.01c	10.16 ± 0.01f	4.09 ± 0.01d
		3.	15.12 ± 0.03e	1.51 ± 0.01e	1.70 ± 0.01e	73.51 ± 0.02b	6.41 ± 0.01e	0.81 ± 0.01d	11.48 ± 0.01d	4.36 ± 0.01b
		4.	12.93 ± 0.06f	1.61 ± 0.01d	1.81 ± 0.02d	75.92 ± 0.04a	5.61 ± 0.01h	0.81 ± 0.02d	13.55 ± 0.02a	5.12 ± 0.02a
**Year**	2010		14.44 ± 0.61c	1.12 ± 0.21c	2.21 ± 0.21a	74.06 ± 1.39a	6.50 ± 0.80c	0.72 ± 0.11c	11.59 ± 1.59a	4.41 ± 0.30a
	2011		14.83 ± 0.55b	1.29 ± 0.15b	1.80 ± 0.14c	72.49 ± 1.56b	7.80 ± 1.47a	0.82 ± 0.11b	9.66 ±2.00c	4.35 ± 0.22b
	2012		15.65 ± 1.21a	1.62 ± 0.37a	1.91 ± 0.20b	72.28 ± 1.95c	6.70 ± 0.89b	0.87 ± 0.14a	11.00 ± 1.63b	4.14 ± 0.46c
	*F*		3595	6805.9	19,674	26,401	22,083	4074	15,546	4854
	*p*		***	***	***	***	***	***	***	***
**Growing site**										
	Kaštela		15.26 ± 0.84a	1.42 ± 0.40a	1.99 ± 0.28a	72.00 ± 1.51b	7.62 ± 1.36a	0.83 ± 0.14a	9.78 ± 1.89b	4.17 ± 0.27b
	Šestanovac		14.69 ± 1.03b	1.28 ± 0.24b	1.95 ± 0.23b	73.88 ± 1.61a	6.38 ± 0.62b	0.77 ± 0.13b	11.72 ± 1.37a	4.44 ± 0.38a
	*F*		2301	1545.2	505	74,587	51,847	1745	44,566	12,907
	*p*		***	***	***	***	***	***	***	***
**Harvest period**										
	1.		15.78 ± 0.83a	1.07 ± 0.17d	2.17 ± 0.29a	71.50 ± 1.42d	7.69 ± 0.64a	0.94 ± 0.1a	9.38 ± 0.92d	3.96 ± 0.26d
	2.		15.27 ± 0.57b	1.31 ± 0.23c	1.97 ± 0.22b	72.71 ± 1.22c	7.08 ± 1.02b	0.82 ± 0.1b	10.47 ± 1.45c	4.19 ± 0.16c
	3.		14.78 ± 0.59c	1.42 ± 0.31b	1.84 ± 0.18d	73.30 ± 1.72b	6.75 ± 1.28c	0.76 ± 0.1c	11.21 ± 1.91b	4.39 ± 0.18b
	4.		14.07 ± 0.96d	1.57 ± 0.38a	1.89 ± 0.17c	74.25 ± 1.74a	6.49 ± 1.51d	0.69 ± 0.1d	11.94 ± 2.20a	4.67 ± 0.33a
	*F*		3746	3573.6	6995	27,905	9032	5884	14,120	16,673
	*p*		***	***	***	***	***	***	***	***

Means marked by different lowercase letters (a–h) in column (for each crop year) and for each main factor (crop year, growing site and harvest period), are significantly different (Tukey’s test, *p* ≤ 0.05). Significance: ***—*p* ≤ 0.001. Harvest period, 1–4, also see Table 1. Values were calculated as the percentage of the total.

**Table 5:** Correlation factors of virgin olive oil fatty acid composition and microclimate parameters (rainfall and mean temperature; Figure 1) in the period of olive fruits intensive growth and ripening.

Parameter	Rainfall					Mean Temperature				
	Jul.	Aug.	Sep.	Oct.	Nov.	Jul.	Aug.	Sep.	Oct.	Nov.
C16:0 ^1^	ns ^2^	−0.413	ns	ns	ns	0.254	0.522	0.328	0.639	ns
C16:1	ns	−0.458	0.228	ns	ns	0.305	0.485	ns	0.299	ns
C17:1	ns	−0.217	0.270	ns	ns	ns	0.268	ns	ns	ns
C18:0	ns	0.208	ns	ns	ns	ns	ns	ns	ns	ns
C18:1	−0.240	0.513	ns	ns	0.424	ns	−0.593	−0.591	−0.533	ns
C18:2	0.235	−0.271	ns	ns	ns	ns	0.291	0.435	ns	ns
C18:3	ns	−0.417	ns	−0.321	ns	ns	0.405	0.342	0.622	ns
C20:0	−0.262	ns	ns	ns	ns	0.287	ns	ns	0.242	ns
C20:1	−0.273	0.246	0.262	ns	ns	0.242	ns	ns	ns	ns
C22:0	ns	ns	ns	ns	ns	ns	ns	ns	ns	−0.411
C24:0	−0.204	ns	ns	ns	ns	0.250	ns	ns	0.432	ns

^1^ C16:0—palmitic acid; C16:1—palmitoleic acid; C17:1—heptadecenoic acid; C18:0—stearic acid; C18:1—oleic acid; C18:2—linoleic acid; C18:3—linolenic acid; C 20:0—arachidonic acid; C20:1—gadoleic acid; C22:0—behenic acid; C24:0—lignoceric acid. ^2^ Statistically significant difference at *p* ≤ 0.05; ns—correlation between parameters not significant *p* ≤ 0.05.

**Table 6:** Correlation factors of virgin olive oil sensory attributes and microclimate parameters (rainfall and mean temperature; Figure 1) in the period of olives’ intensive growth and ripening.

Parameter	Rainfall					Mean Temperature				
	Jul.	Aug.	Sep.	Oct.	Nov.	Jul.	Aug.	Sep.	Oct.	Nov.
Fruity ^1^	ns ^2^	0.233	ns	−0.4005	0.373	ns	−0.274	−0.3400	ns	ns
Bitter	ns	0.197	−0.3333	−0.371	0.572	−0.382	−0.582	−0.298	−0.3418	−0.3108
Pungent	ns	0.259	−0.150	−0.463	0.311	−0.2629	−0.416	−0.220	ns	ns
Sweet	ns	ns	0.297	0.345	−0.5101	0.319	0.445	0.187	0.200	0.485
Astrig.	0.192	ns	−0.335	−0.216	0.493	−0.291	−0.296	ns	−0.199	−0.290
Green	ns	0.380	ns	−0.571	ns	−0.188	−0.4403	−0.328	ns	ns
Apple	0.291	−0.412	ns	ns	ns	ns	0.182	0.297	0.181	ns
Almond	−0.2216	ns	ns	ns	0.474	0.155	ns	−0.360	ns	ns
OQS	−0.220	0.268	0.205	−0.475	ns	ns	ns	−0.259	ns	ns

^1^ Identification: Astrig—astringent, OQS—overall quality score; ^2^ statistically significant difference at *p* ≤ 0.05, ns—correlation between parameters not significant *p* ≤ 0.05.

## Data Availability

The original contributions generated for this study are included in the article/Supplementary Material; further inquiries can be directed to the corresponding author.

## Supplementary Materials

The following are available online at https://www.mdpi.com/article/10.3390/antiox10050689/s1, Table S1. Basic quality parameters of Oblica virgin olive oils during ripening obtained from two distinct olive orchards (Kaštela and Šestanovac) in three successive crop years; Table S2. Basic quality parameters of Leccino virgin olive oils during ripening obtained from two distinct olive orchards (Kaštela and Šestanovac) in three successive crop years; Table S3. Less represented fatty acids and the percentage of total saturated, monounsaturated, and polyunsaturated fatty acids of Oblica virgin olive oils during ripening, obtained from two distinct olive orchards (Kaštela and Šestanovac) in three successive crop years; Table S4. Less represented fatty acids and the percentage of total saturated, monounsaturated, and polyunsaturated fatty acids of Leccino virgin olive oils during ripening, obtained from two distinct olive orchards (Kaštela and Šestanovac) in three successive crop years; Table S5. Results of three-way analysis of variance for sensory properties of Oblica virgin olive oils, obtained from two distinct olive orchards in three successive crop years; Table S6. Results of three-way analysis of variance for sensory properties of Leccino virgin olive oils, obtained from two distinct olive orchards in three successive crop years; Figure S1. Overall quality score of A) Oblica and B) Leccino virgin olive oils, obtained from two distinct olive orchards in three successive crop years, 2010, 2011 and 2012 respectively.
