# Supplementary material for: Virgin Olive Oil Phenols, Fatty Acid Composition and Sensory Profile: Can Cultivar Overpower Environmental and Ripening Effect?
Source: Antioxidants (Basel). 2021 Apr 27;10(5):689. doi: 10.3390/antiox10050689 (PMC8144995; doi:10.3390/antiox10050689)
Supplement: Supplementary file 1 [file antioxidants-10-00689-s001.zip › antioxidants-1182028-supplementary.pdf]

antioxidants

MDPI

SUPPLEMENTARY MATERIAL

Supplemental Table 1. Basic quality parameters of Oblica virgin olive oils during ripening obtained from two distinct olive orchards (Kaštela and Šestanovac) in three successive crop years

Factor			FFA	PV	K_{232}	K_{270}
2010	Kaštela	1.	0.32±0.03ab	3.6±0.2de	1.62±0.01c	0.15±0.02ab
		2.	0.38±0.03a	4.2±0.2cd	1.56±0.02c	0.11±0.02b
		3.	0.33±0.05ab	6.6±0.2a	1.88±0.03a	0.21±0.02a
		4.	0.33±0.01ab	6.6±0.2a	1.85±0.01ab	0.21±0.01a
	Šestanovac	1.	0.27±0.03bc	4.6±0.2bc	1.67±0.05c	0.14±0.01ab
		2.	0.26±0.01bc	2.6±0.2f	1.54±0.07c	0.10±0.04b
		3.	0.35±0.03ab	5.2±0.4b	1.70±0.01bc	0.21±0.01a
		4.	0.18±0.02c	3.2±0.2ef	1.64±0.08c	0.15±0.06ab
2011	Kaštela	1.	0.31±0.02b	6.2±0.2a	1.78±0.03de	0.17±0.03b
		2.	0.24±0.01c	4.0±0.2de	1.86±0.02cde	0.21±0.01a
		3.	0.25±0.01c	4.6±0.2cd	1.73±0.08e	0.16±0.01b
		4.	0.31±0.01b	5.6±0.6ab	2.08±0.09ab	0.18±0.01ab
	Šestanovac	1.	0.38±0.01a	5.2±0.2bc	1.96±0.01bcd	0.22±0.01a
		2.	0.36±0.02a	3.6±0.2e	2.15±0.02a	0.21±0.01a
		3.	0.38±0.01a	5.8±0.2ab	2.02±0.05abc	0.21±0.01a
		4.	0.26±0.01c	5.6±0.4ab	2.08±0.01ab	0.21±0.01a
2012	Kaštela	1.	0.23±0.03b	4.4±0.2b	1.65±0.04d	0.15±0.02ab
		2.	0.22±0.01b	3.6±0.2cd	1.61±0.07d	0.17±0.05ab
		3.	0.22±0.02b	3.8±0.2c	1.53±0.01d	0.10±0.02b
		4.	0.32±0.01a	2.6±0.2 f	1.44±0.06 e	0.11±0.01b
	Šestanovac	1.	0.32±0.01a	3.2±0.2e	2.17±0.01a	0.22±0.01a
		2.	0.23±0.01b	7.2±0.2a	2.12±0.05a	0.20±0.02a
		3.	0.34±0.01a	4.2±0.2b	1.95±0.03b	0.17±0.01a
		4.	0.31±0.01a	3.4±0.2de	1.72±0.02c	0.22±0.01a
Crop Year						
		2010	0.30±0.07a	4.44±1.44b	1.68±0.13b	0.16±0.05b
		2011	0.31±0.06a	4.98±0.86a	1.96±0.16a	0.20±0.03a
		2012	0.27±0.06b	3.98±1.36c	1.76±0.45b	0.17±0.05b
		F	*20.57*	*181.10*	*253.42*	*16.196*
		p	***	***	***	***
Growing area						
		Kaštela	0.29±0.06b	4.54±1.26a	1.72±0.19b	0.16±0.04b
		Šestanovac	0.30±0.07a	4.38±1.34b	1.89±0.42a	0.19±0.05a
		F	*6.93*	*14.14*	*63.13*	*23.736*
		p	*	***	***	***
Harvest period						
		1.	0.31±0.06a	4.40±1.04b	1.81±0.21a	0.18±0.04
		2.	0.28±0.07b	4.12±1.50c	1.81±0.28a	0.17±0.06
		3.	0.31±0.07a	4.92±0.98a	1.80±0.18a	0.18±0.05
		4.	0.29±0.06b	4.40±1.56b	1.76±0.29b	0.18±0.05
		F	*9.59*	*57.81*	*37.76*	*1.196*
		p	***	***	***	ns

Means marked by different lowercase letters in column (for each crop year) and for each main factor (crop year, growing area and harvest period), are significantly different (Tukey's test, $p \leq 0.05$), ns – not significant. 1–4, harvest period, see also Table 1. Identification: FFA – free fatty acid (% oleic acid); PV – peroxide value (meq O_2/kg). *** *median of olive fruity in all samples was > 0; median of sensory defect in all samples was < 0.*

Supplemental Table 2. Basic quality parameters of Leccino virgin olive oils during ripening obtained from two distinct olive orchards (Kaštela and Šestanovac) in three successive crop years

Factor			FFA	PV	K_{232}	K_{270}
2010	Kaštela	1.	0.36±0.04ab	0.15±0.02ab	1.72±0.01e	0.18±0.02b
		2.	0.35±0.01ab	0.11±0.02b	1.80±0.02d	0.21±0.01a
		3.	0.31±0.02b	0.21±0.02a	1.50±0.02f	0.13±0.01c
		4.	0.31±0.01b	0.21±0.01a	1.78±0.01d	0.22±0.01a
	Šestanovac	1.	0.32±0.02ab	0.14±0.01ab	1.97±0.02b	0.17±0.02b
		2.	0.30±0.02b	0.10±0.04b	1.91±0.01c	0.21±0.01a
		3.	0.37±0.02a	0.21±0.01a	2.02±0.03a	0.17±0.01b
		4.	0.18±0.02c	0.15±0.06ab	1.71±0.01e	0.12±0.01c
2011	Kaštela	1.	0.39±0.03a	0.17±0.03b	1.72±0.03c	0.14±0.03a
		2.	0.42±0.02a	0.21±0.01a	1.72±0.05c	0.19±0.05a
		3.	0.24±0.01c	0.16±0.01b	1.83±0.03b	0.16±0.05a
		4.	0.24±0.02c	0.18±0.01ab	1.79±0.03bc	0.15±0.01a
	Šestanovac	1.	0.40±0.01a	0.22±0.01a	2.05±0.01a	0.21±0.01a
		2.	0.32±0.02b	0.21±0.01a	1.97±0.01a	0.21±0.02a
		3.	0.35±0.02ab	0.21±0.01a	1.97±0.02a	0.21±0.01a
		4.	0.25±0.02c	0.21±0.01a	1.77±0.01bc	0.12±0.01a
2012	Kaštela	1.	0.21±0.01c	0.15±0.02ab	1.58±0.01a	0.17±0.01a
		2.	0.21±0.02c	0.17±0.05ab	1.51±0.07a	0.13±0.03ab
		3.	0.23±0.01b	0.10±0.02b	1.79±0.17a	0.12±0.02b
		4.	0.35±0.01a	0.11±0.01b	1.64±0.04a	0.14±0.03abc
	Šestanovac	1.	0.24±0.03bc	0.22±0.01a	1.52±0.03a	0.16±0.01ab
		2.	0.24±0.02bc	0.20±0.02a	1.68±0.04a	0.17±0.02a
		3.	0.29±0.03b	0.17±0.01a	1.64±0.07a	0.14±0.02abc
		4.	0.38±0.02a	0.22±0.01a	1.88±0.06a	0.16±0.01ab
Crop Year						
	2010		0.31±0.06a	5.84±1.44b	1.80±0.17a	0.18±0.04a
	2011		0.32±0.08a	6.50±1.16a	1.85±0.13a	0.17±0.04a
	2012		0.27±0.07b	5.02±1.84c	1.65±0.39b	0.14±0.03b
	F		*61.73*	*102.72*	*32.721*	*22.632*
	p		***	***	***	***
Growing area						
	Kaštela		0.30±0.08	5.66±1.92b	1.69±0.43b	0.16±0.04
	Šestanovac		0.30±0.07	5.90±1.24a	1.83±0.38a	0.16±0.04
	F		*0.10*	*8.27*	*12.275*	*0.394*
	p		ns	***	***	ns
Harvest period						
	1.		0.32±0.08a	6.50±1.08a	1.76±0.21a	0.17±0.03ab
	2.		0.31±0.08ab	6.08±1.14b	1.77±0.17a	0.18±0.06a
	3.		0.30±0.06bc	5.12±1.66c	1.72±0.12b	0.15±0.04b
	4.		0.29±0.08c	5.42±2.1c	1.77±0.21a	0.15±0.04b
	F		*9.30*	*55.10*	*7.121*	*5.887*
	p		***	***	**	***

Means marked by different lowercase letters in column (for each crop year) and for each main factor (crop year, growing area and harvest period), are significantly different (Tukey's test, p ≤ 0.05), ns – not significant. 1–4, harvest period, see also Table 1. Identification: FFA – free fatty acid (% oleic acid); PV – peroxide value (meq O_2/kg); *** median of olive fruity in all samples was > 0; median of sensory defect in all samples was < 0.

antioxidants

MDPI

Supplemental Table 3. Less represented fatty acids and the percentage of total saturated, monounsaturated, and polyunsaturated fatty acids of Oblica virgin olive oils during ripening obtained from two distinct olive orchards (Kaštela and Šestanovac) in three successive crop years

Factor			Fatty acid								
			C17:1	C20:0	C20:1	C22:0	C24:0	SFA	PUFA	MUFA	MUFA/PUFA
2010	Kaštela	1.	0.11±0.01	0.51±0.01a	0.41±0.01	0.11±0.01	0.10±0.01	17.13±0.03a	10.51±0.02e	72.45±0.03g	6.90±0.01d
		2.	0.11±0.01	0.51±0.01a	0.41±0.01	0.11±0.01	0.10±0.01	16.65±0.12b	11.01±0.02b	72.43±0.03g	6.58±0.01g
		3.	0.11±0.01	0.41±0.01b	0.41±0.01	0.11±0.01	0.11±0.01	15.72±0.02c	10.82±0.03c	73.54±0.03f	6.81±0.02e
		4.	0.11±0.01	0.41±0.01b	0.41±0.01	0.11±0.01	0.11±0.01	14.74±0.02e	11.13±0.02a	74.12±0.02e	6.67±0.01f
	Šestanovac	1.	0.11±0.01	0.51±0.01a	0.41±0.01	0.11±0.01	0.11±0.01	15.74±0.02c	9.23±0.03h	75.12±0.02c	8.15±0.03a
		2.	0.11±0.01	0.41±0.01b	0.41±0.01	0.11±0.01	0.11±0.01	15.23±0.04d	10.21±0.01g	74.63±0.03d	7.32±0.01b
		3.	0.10±0.01	0.41±0.01b	0.41±0.01	0.11±0.01	0.11±0.01	13.55±0.04f	10.41±0.01f	76.03±0.03b	7.31±0.01b
		4.	0.11±0.01	0.41±0.01b	0.41±0.01	0.11±0.01	0.11±0.01	12.72±0.03g	10.62±0.03d	77.06±0.05a	7.26±0.02c
2011	Kaštela	1.	0.10±0.01	0.41±0.01	0.31±0.01b	0.10±0.01	0.11±0.01	17.42±0.04c	15.10±0.02c	67.51±0.01f	4.48±0.01f
		2.	0.10±0.01	0.41±0.01	0.31±0.01b	0.11±0.01	0.10±0.01	18.02±0.02a	15.31±0.01a	66.61±0.01h	4.36±0.01h
		3.	0.11±0.01	0.41±0.01	0.31±0.01b	0.11±0.01	0.11±0.01	17.61±0.03b	15.21±0.01b	67.21±0.01g	4.43±0.01g
		4.	0.11±0.01	0.41±0.01	0.41±0.01a	0.11±0.01	0.11±0.01	16.41±0.01d	15.01±0.01d	68.42±0.03e	4.57±0.01e
	Šestanovac	1.	0.11±0.01	0.41±0.01	0.31±0.01b	0.11±0.01	0.10±0.01	17.22±0.02e	12.01±0.01e	70.61±0.01d	5.89±0.01d
		2.	0.11±0.01	0.41±0.01	0.31±0.02b	0.11±0.01	0.11±0.01	16.51±0.01g	11.91±0.01g	71.52±0.02c	6.01±0.01b
		3.	0.11±0.01	0.41±0.01	0.31±0.01b	0.11±0.01	0.11±0.01	15.81±0.02f	11.81±0.01h	72.31±0.01b	6.13±0.01a
		4.	0.11±0.01	0.41±0.01	0.31±0.01b	0.10±0.01	0.11±0.01	15.21±0.01h	12.21±0.02f	72.61±0.01a	5.95±0.01c
2012	Kaštela	1.	0.11±0.01	0.51±0.01a	0.41±0.01a	0.11±0.01	0.10±0.01	18.22±0.06a	12.72±0.04d	69.11±0.05g	5.44±0.03
		2.	0.11±0.01	0.51±0.01a	0.41±0.01a	0.11±0.01	0.10±0.01	17.81±0.03b	13.01±0.03c	69.24±0.03fg	5.33±0.01
		3.	0.10±0.01	0.51±0.01a	0.41±0.01a	0.11±0.01	0.11±0.01	17.23±0.07c	14.51±0.11a	69.41±0.01e	4.79±0.04
		4.	0.11±0.01	0.51±0.01a	0.41±0.01a	0.10±0.01	0.11±0.01	16.61±0.01d	14.11±0.03b	69.31±0.02ef	4.92±0.01
	Šestanovac	1.	0.11±0.01	0.51±0.01a	0.31±0.01b	0.10±0.01	0.11±0.01	17.70±0.06c	11.01±0.01e	71.42±0.08d	6.50±0.01
		2.	0.10±0.01	0.41±0.01b	0.31±0.01b	0.11±0.01	0.11±0.01	16.63±0.05d	10.51±0.04f	72.81±0.09c	6.94±0.02
		3.	0.11±0.01	0.41±0.01b	0.31±0.01b	0.11±0.01	0.11±0.01	15.63±0.04e	10.51±0.01f	73.81±0.07b	7.03±0.01
		4.	0.11±0.01	0.41±0.01b	0.31±0.01b	0.11±0.01	0.11±0.01	14.73±0.03f	9.92±0.04g	75.43±0.04a	7.62±0.03

Supplemental Table 3. (continued)

Factor		Fatty acid								
		C17:1	C20:0	C20:1	C22:0	C24:0	SFA	PUFA	MUFA	MUFA/PUFA
Main effects										
Crop Year										
	2010	0.11±0.01	0.44±0.05b	0.41±0.01a	0.11±0.01	0.11±0.01	15.19±1.43c	10.49±0.57c	74.42±1.57a	7.13±0.49a
	2011	0.11±0.01	0.41±0.01c	0.32±0.04c	0.11±0.01	0.11±0.01	16.77±0.92b	13.57±1.63a	69.60±2.33c	5.23±0.80c
	2012	0.11±0.01	0.47±0.05a	0.36±0.06b	0.11±0.01	0.11±0.01	16.82±1.13a	12.04±1.7b	71.32±2.35b	6.07±1.04b
	F	*0.062*	*11261.0*	*4077.6*	*0.466*	*1.414*	*14558*	*69621*	*205993*	*133189*
	p	*ns*	***	***	*ns*	*ns*	***	***	***	***
Growing site										
	Kaštela	0.11±0.01	0.46±0.06a	0.38±0.05a	0.11±0.01	0.11±0.01	16.96±0.98a	13.20±1.86a	69.95±2.48b	5.44±0.99b
	Šestanovac	0.11±0.01	0.42±0.04b	0.34±0.05b	0.11±0.01	0.11±0.01	15.56±1.39b	10.86±0.91b	73.61±1.99a	6.84±0.72a
	F	*0.423*	*9153.0*	*2817.7*	*0.135*	*0.131*	*25042*	*121100*	*122194*	*217666*
	p	*ns*	***	***	*ns*	*ns*	***	***	***	***
Harvest period										
	1.	0.11±0.01	0.47±0.05a	0.36±0.06b	0.11±0.01	0.11±0.01	17.24±0.79a	11.76±1.92d	71.04±2.49d	6.23±1.19a
	2.	0.11±0.01	0.44±0.05b	0.36±0.06b	0.11±0.01	0.11±0.01	16.81±0.95b	11.99±1.81c	71.20±2.69c	6.09±1.04c
	3.	0.11±0.01	0.42±0.04c	0.36±0.06b	0.11±0.01	0.11±0.01	15.93±1.35c	12.21±2.00a	72.05±3.02b	6.08±1.14c
	4.	0.11±0.01	0.42±0.04c	0.37±0.05a	0.11±0.01	0.11±0.01	15.07±1.33d	12.17±1.9b	72.83±3.21a	6.16±1.17b
	F	*0.621*	*4564.0*	*108.8*	*0.422*	*1.563*	*11747*	*911*	*10481*	*502*
	p	*ns*	***	***	*ns*	*ns*	***	***	***	***

Means marked by different lowercase letters in column (for each crop year) and for each main factor (crop year, growing area and harvest period), are significantly different (Tukey's test, $p \leq 0.05$), ns – not significant. 1–4, harvest period, see also Table 1. Values were calculated as the percentage of the total.

Supplemental Table 4. Less represented fatty acids and the percentage of total saturated, monounsaturated, and polyunsaturated fatty acids of Leccino virgin olive oils during ripening obtained from two distinct olive orchards (Kaštela and Šestanovac) in three successive crop years

Factor			Fatty acid								
			C17:1	C20:0	C20:1	C22:0	C24:0	SFA	PUFA	MUFA	MUFA/PUFA
2010	Kaštela	1.	0.10±0.00	0.41±0.01a	0.30±0.00	0.11±0.01	0.10±0.00	18.37±0.04a	8.82±0.02a	73.04±0.06g	8.29±0.02g
		2.	0.10±0.00	0.41±0.01a	0.30±0.00	0.11±0.01	0.10±0.00	17.91±0.01b	7.91±0.01b	74.22±0.02f	9.40±0.01f
		3.	0.10±0.00	0.31±0.01b	0.30±0.00	0.11±0.01	0.10±0.00	17.11±0.01c	7.31±0.01c	75.72±0.03e	10.38±0.01e
		4.	0.10±0.00	0.31±0.01b	0.30±0.00	0.11±0.01	n.d.	16.61±0.01d	6.62±0.02e	76.64±0.06c	11.59±0.02c
	Šestanovac	1.	n.d.	0.31±0.01b	0.30±0.00	0.11±0.01	0.10±0.00	17.92±0.02b	7.91±0.02b	74.22±0.02f	9.39±0.02f
		2.	0.10±0.00	0.41±0.01a	0.30±0.00	0.11±0.01	0.10±0.00	17.21±0.01c	6.82±0.02d	76.16±0.06d	11.19±0.04d
		3.	0.10±0.00	0.31±0.01b	0.30±0.00	0.11±0.01	0.10±0.00	16.63±0.03d	6.21±0.01f	77.32±0.03a	12.47±0.01b
		4.	0.10±0.00	0.31±0.01b	0.20±0.00	0.11±0.01	n.d.	15.81±0.01e	6.13±0.04g	77.11±0.01b	12.59±0.07a
2011	Kaštela	1.	0.10±0.00	0.31±0.01	0.31±0.02	0.11±0.01	0.10±0.00	17.42±0.02c	9.35±0.05d	73.36±0.02e	7.85±0.04e
		2.	0.10±0.00	0.31±0.01	0.31±0.01	0.11±0.01	0.10±0.00	17.61±0.03b	10.03±0.01c	72.91±0.01g	7.28±0.01f
		3.	0.10±0.00	0.31±0.01	0.31±0.01	0.10±0.01	0.10±0.00	17.02±0.02d	10.34±0.05b	71.81±0.01h	6.96±0.04g
		4.	0.10±0.00	0.31±0.01	0.31±0.01	0.11±0.01	0.10±0.00	16.53±0.02f	10.54±0.02a	73.03±0.03f	6.94±0.02g
	Šestanovac	1.	0.10±0.00	0.31±0.01	0.31±0.01	0.11±0.01	0.10±0.00	18.02±0.02a	7.73±0.03e	74.32±0.03d	9.63±0.03d
		2.	0.10±0.00	0.31±0.02	0.31±0.01	0.11±0.01	n.d.	17.43±0.03c	7.02±0.02g	75.53±0.02b	10.78±0.02b
		3.	0.10±0.00	0.31±0.01	0.31±0.01	0.11±0.01	n.d.	16.61±0.01e	7.21±0.01f	75.25±0.02c	10.45±0.02c
		4.	0.10±0.00	0.31±0.02	0.31±0.01	0.11±0.01	n.d.	16.13±0.02g	6.72±0.01h	77.21±0.01a	11.50±0.01a
2012	Kaštela	1.	0.10±0.00	0.41±0.01a	0.31±0.01	0.11±0.01	0.10±0.00	20.03±0.22a	9.52±0.02a	70.67±0.07e	7.43±0.02h
		2.	0.10±0.00	0.31±0.04b	0.31±0.02	0.11±0.01	0.10±0.00	18.52±0.06c	7.61±0.03d	73.82±0.06c	9.71±0.05e
		3.	0.10±0.00	0.31±0.01b	0.31±0.01	0.11±0.01	0.10±0.00	18.13±0.03d	6.74±0.06f	75.23±0.05b	11.17±0.10c
		4.	0.10±0.00	0.31±0.02b	0.31±0.01	0.11±0.01	0.10±0.00	18.12±0.02d	6.61±0.04g	75.32±0.02b	11.41±0.07b
	Šestanovac	1.	0.10±0.00	0.41±0.01a	0.31±0.01	0.11±0.01	0.10±0.00	19.31±0.06b	8.41±0.02b	72.1±0.07d	8.58±0.02g
		2.	0.10±0.00	0.31±0.01b	0.31±0.01	0.10±0.00	0.10±0.00	18.11±0.02d	8.01±0.01c	73.93±0.05c	9.24±0.01f
		3.	0.10±0.00	0.31±0.01b	0.31±0.01	0.11±0.01	0.10±0.00	17.32±0.04e	7.21±0.01e	75.42±0.02b	10.47±0.01d
		4.	0.10±0.00	0.31±0.01b	0.31±0.01	0.11±0.01	n.d.	15.24±0.06f	6.41±0.02h	77.93±0.04a	12.17±0.03a

Supplemental Table 4. (continued)

Factor		Fatty acid								
		C17:1	C20:0	C20:1	C22:0	C24:0	SFA	PUFA	MUFA	MUFA/PUFA
Main effects										
Crop Year										
	2010	0.08±0.03b	0.34±0.05a	0.29±0.04b	0.11±0.01	0.07±0.4b	17.2±0.81b	7.21±0.91c	75.55±1.50a	10.66±1.49a
	2011	0.10±0.01a	0.31±0.01c	0.31±0.01a	0.11±0.01	0.06±0.05c	17.1±0.61c	8.62±1.54a	74.18±1.68c	8.92±1.79c
	2012	0.11±0.01a	0.33±0.05b	0.31±0.01a	0.11±0.01	0.08±0.03a	18.1±1.37a	7.56±1.01b	74.3±2.13b	10.02±1.52b
	F	*3253*	*116.0*	*86.6*	*o.308*	*12795*	*2825*	*22353*	*11608*	*16536*
	p	***	***	***	ns	***	***	***	***	***
Growing site										
	Kaštela	0.10±0.00a	0.33±0.05a	0.31±0.01a	0.11±0.01	0.09±0.03a	17.78±0.95a	8.45±1.45a	73.81±1.67b	9.03±1.75b
	Šestanovac	0.09±0.03b	0.32±0.04b	0.3±0.03b	0.11±0.01	0.06±0.05b	17.15±1.1b	7.15±0.72b	75.54±1.67a	10.70±1.29a
	F	*3270*	*19.5*	*77.8*	*0.052*	*65780*	*2815*	*53144*	*45033*	*44642*
	p	***	***	***	ns	***	***	***	***	***
Harvest period										
	1.	0.08±0.04b	0.36±0.06a	0.31±0.01a	0.11±0.01	0.10±0.00c	18.51±0.93a	8.62±0.70a	72.95±1.31d	8.53±0.81d
	2.	0.10±0.00a	0.34±0.05b	0.31±0.01a	0.11±0.01	0.08±0.04a	17.80±0.45b	7.90±1.08b	74.43±1.13c	9.60±1.30c
	3.	0.10±0.00a	0.31±0.01c	0.31±0.01a	0.11±0.01	0.08±0.04a	17.14±0.53c	7.50±1.37c	75.12±1.70b	10.32±1.72b
	4.	0.10±0.00a	0.31±0.01c	0.29±0.04b	0.11±0.01	0.03±0.05b	16.41±0.92d	7.17±1.57d	76.2±1.68a	11.03±1.94a
	F	*3251*	*163.1*	*73.2*	*0.185*	*50064*	*5666*	*12291*	*28018*	*18261*
	p	***	***	***	ns	***	***	***	***	***

Means marked by different lowercase letters in column (for each crop year) and for each main factor (crop year, growing area and harvest period), are significantly different (Tukey's test, p ≤ 0.05), ns – not significant. 1–4, harvest period, see also Table 1. Values were calculated as the percentage of the total.

antioxidants

Supplemental Table 5. Results of three-way analysis of variance for sensory properties of Oblica virgin olive oils obtained from two distinct olive orchards in three successive crop years

Factor	Sensory attributes							
	Fruity	Bitter	Pungent	Sweet	Astringent	Green	Apple	Almond
Crop Year								
2010	5.74±1.3a	5.32±1.56b	6.46±1.53a	1.91±1.62ab	2.30±1.02b	4.76±1.78a	1.88±1.00b	2.86±0.96
2011	5.90±1.1a	5.82±1.54a	6.14±0.79a	1.84±0.94b	3.29±2.08a	4.76±1.18a	3.30±0.97a	3.09±0.94
2012	5.26±1.05b	5.21±2.62b	5.47±1.13b	2.38±1.52a	3.75±2.18a	3.94±1.42b	3.03±0.70a	3.02±1.58
F	*8.541*	*15.830*	*9.058*	*6.356*	*39.928*	*5.609*	*39.375*	*2.425*
p	***	***	***	***	***	***	***	*ns*
Growing area								
Kaštela	5.27±1.27b	4.26±1.68b	5.56±1.26b	2.58±1.76a	2.19±1.00b	4.18±1.58b	2.91±0.98a	2.36±1.07b
Šestanovac	6.00±0.96a	6.55±1.40a	6.50±1.05a	1.52±0.60b	3.88±2.13a	4.83±1.40a	2.55±1.18b	3.55±0.93a
F	*26.593*	*312.305*	*19.825*	*55.321*	*154.692*	*8.391*	*2.981*	*68.176*
p	***	***	***	***	***	***	ns	***
Harvest period								
1.	6.12±1.27a	5.94±1.78a	6.33±1.12a	1.41±0.65c	3.88±1.99a	5.06±1.42a	3.14±1.06a	3.28±1.21
2.	5.6±1.05ab	5.78±1.95ab	6.22±1.00a	1.37±0.75c	3.33±2.29b	4.87±1.30a	2.46±1.02b	2.81±1.13
3.	5.74±1.20a	5.25±2.00bc	6.27±1.28a	2.24±1.05b	2.82±1.53b	4.80±1.59a	3.07±0.96a	2.95±0.98
4.	5.09±1.01b	4.77±1.91c	5.30±1.40b	3.25±1.96a	2.14±1.16c	3.18±1.01b	2.17±1.16b	2.89±1.36
F	*12.250*	*20.677*	*8.650*	*41.588*	*16.876*	*15.542*	*9.186*	*2.060*
p	***	***	***	***	***	***	***	*ns*

Means marked by different lowercase letters in column for each main factor (crop year, growing area and harvest period), are significantly different (Tukey's test, $p \le 0.05$). 1–4, harvest period, see also Table 1. Ns – not significant.

Supplemental Table 6. Results of three-way analysis of variance for sensory properties of Leccino virgin olive oils obtained from two distinct olive orchards in three successive crop years

Factor	Sensory attributes							
	**Fruity**	**Bitter**	**Pungent**	**Sweet**	**Astringent**	**Green**	**Apple**	**Almond**
Crop Year								
2010	5.97±1.57a	6.67±2.09a	5.78±1.32a	2.17±1.65b	2.74±1.38a	5.56±1.52a	2.32±1.06a	3.1±1.03b
2011	3.93±1.32c	3.95±2.44b	4.43±1.63b	2.98±1.53a	2.17±2.24a	2.86±1.29c	2.37±0.83a	1.63±0.83c
2012	5.29±0.81b	3.34±2.04c	4.24±1.29b	3.09±1.56a	1.68±1.07b	3.6±0.75b	2.39±0.66a	3.89±0.98a
F	48.264	155.748	24.481	5.201	19.635	139.072	1.663	77.028
p	***	***	***	***	***	***	*ns*	***
Growing area								
Kaštela	4.69±1.55b	3.23±2.24b	4.34±1.48b	3.71±1.39a	1.23±0.75b	3.37±1.45b	2.18±0.82b	2.67±1.43b
Šestanovac	5.55±1.38a	6.22±2.08a	5.37±1.48a	1.74±1.17b	3.2±1.72a	4.79±1.61a	2.54±0.87a	3.16±1.16a
F	40.499	385.779	35.357	99.508	218.484	109.522	9.058	14.838
p	***	***	***	***	***	***	**	***
Harvest period								
1.	5.03±1.48c	4.18±2.64c	4.53±1.36c	3.06±1.61a	2.37±1.8a	4.24±1.45b	1.8±0.79b	3.24±1.49a
2.	6.15±1.61a	6.16±2.52a	5.86±1.27a	2.48±1.52b	2.49±1.67a	5.22±1.57a	2.96±0.44a	3.29±1.11a
3.	5.42±1.00b	5.35±2.24b	5.31±1.28b	1.84±1.46b	2.51±1.77a	4.00±1.52b	2.57±0.90a	3.11±1.23a
4.	3.95±1.18d	3.25±2.33d	3.75±1.53d	3.59±1.42a	1.53±1.24b	3.02±1.53c	2.06±0.79b	2.10±1.16b
F	31.690	54.668	29.524	13.040	13.841	46.713	18.305	15.074
p	***	***	***	***	***	***	***	***

Means marked by different lowercase letters in column for each main factor (crop year, growing area and harvest period), are significantly different (Tukey's test, $p \leq 0.05$). 1–4, harvest period, see also Table 1. Ns – not significant.

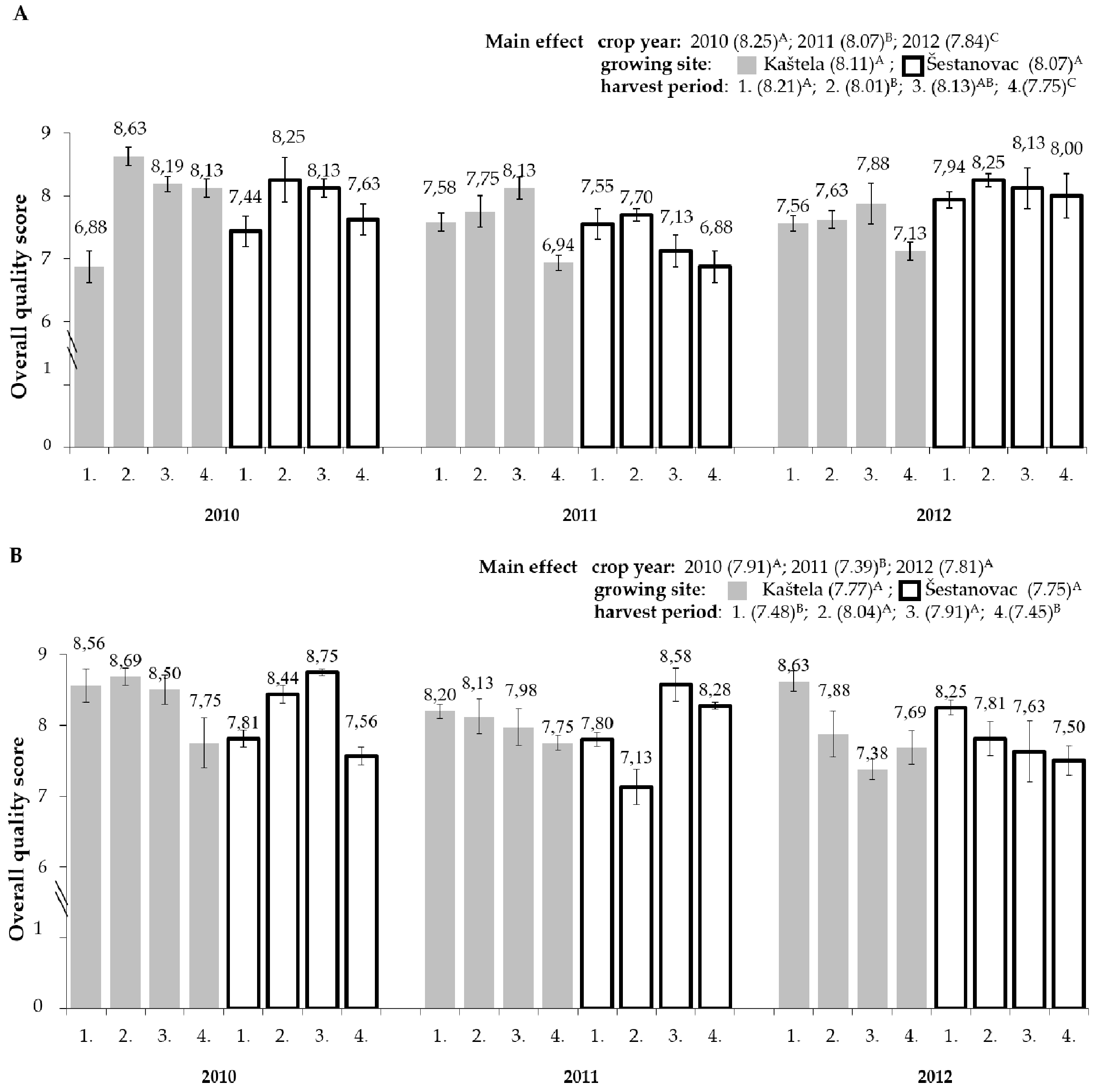

Supplemetal Figure 1. Overall quality score of A) Oblica and B) Leccino virgin olive oils obtained from two distinct olive orchards in three successive crop years, 2010, 2011 and 2012 respectively. Different uppercase letters indicate differences within main effects (crop year, growing area and harvest period) obtained by three-way ANOVA (Tukey's test, $p \leq 0.05$). 1–4, harvest period, see also Table 1.
